# Characterization of Pathogenic and Nonpathogenic *Fusarium oxysporum* Isolates Associated with Commercial Tomato Crops in the Andean Region of Colombia

**DOI:** 10.3390/pathogens9010070

**Published:** 2020-01-20

**Authors:** Sandra L. Carmona, Diana Burbano-David, Magda R. Gómez, Walter Lopez, Nelson Ceballos, Jairo Castaño-Zapata, Jaime Simbaqueba, Mauricio Soto-Suárez

**Affiliations:** 1 Corporación Colombiana de Investigación Agropecuaria. AGROSAVIA, Km 14 vía Mosquera-Bogotá, Mosquera 250047, Colombia; scarmona@agrosavia.co (S.L.C.); dmburbano@agrosavia.co (D.B.-D.); mrgomez@agrosavia.co (M.R.G.); jsimbaqueba@gmail.com (J.S.); 2 Departamento de Física y Química, Facultad de Ciencias Exactas y Naturales, Universidad Nacional de Colombia sede Manizales, Manizales 170004, Colombia; wrlopez@unal.edu.co; 3 Facultad de Ciencias Agropecuarias, Universidad de Caldas, Manizales 170004, Colombia; nelson.ceballos@ucaldas.edu.co (N.C.); jairo.castano_z@ucaldas.edu.co (J.C.-Z.)

**Keywords:** *Fusarium* wilt, physiological races, *SIX* genes, virulence, *forma specialis*, *Solanaceae*

## Abstract

In Colombia, tomato production under protected conditions represents an important economic contribution to the agricultural sector. *Fusarium* wilt diseases, caused by pathogenic *formae speciales* of the soil-borne fungus *Fusarium oxysporum* Schltdl., cause significant yield losses in tomatoes throughout the world. Investigation of the *F. oxysporum*–tomato pathosystem in Colombia is required to develop appropriate alternative disease management. In this study, 120 fungal isolates were obtained from four different departments in the Central Andean Region in Colombia from tomato crops with symptoms of wilt disease. A molecular characterization of the fungal isolates was performed using the *SIX1*, *SIX3*, and *SIX4* effector genes of *Fusarium oxysporum* f. sp. *lycopersici* W.C. Snyder & H.N. Hansen (*Fol*). Additionally, we developed a new specific marker to distinguish between *Fusarium oxysporum* f. sp. *radicis-lycopersici* Jarvis & Shoemaker (*Forl*) and *Fol* isolates. Furthermore, a phylogenetic analysis using the *Translation Elongation Factor 1-alpha* (*EF1a*) gene was performed with the collected isolates. Two isolates (named *Fol*59 and *Fol*-UDC10) were identified as *Fol* race 2, four isolates were identified as *Forl*, six isolates were identified as *F. solani*, and most of the isolates were grouped within the *F. oxysporum* species complex. The phylogenetic tree of *EF1a* showed that most of the isolates could potentially correspond to nonpathogenic strains of *F. oxysporum*. Additional pathogenicity assays carried out with *Fol*59 and *Fol-*UDC10 confirmed that both isolates were highly virulent strains. This study represents a contribution to the understanding of the local interaction between tomatoes and *F. oxysporum* in Colombia.

## 1. Introduction

*Fusarium oxysporum* is a ubiquitous species complex of fungi that includes soil-borne plant pathogenic lineages that are the causal agents of vascular wilt disease in a broad range of plant species, such as bananas, cotton, tomatoes, and legumes [1,2,3,4]. Pathogenic strains of *F. oxysporum* are grouped into *formae speciales* (ff. spp.), depending on the host species they infect [5,6,7]. A *forma specialis* (f. sp.) can be subdivided into races that are defined by the virulence patterns on the resistant or susceptible varieties of the host species [3].

Tomatoes are one of the most important vegetable crops worldwide [8]. However, their production is severely affected by diseases caused by pathogens. Among these, vascular wilt and crown root rot diseases caused by *Fusarium oxysporum* ff. spp. *lycopersici* (*Fol*) and *radicis-lycopersici* (*Forl*), respectively, are responsible for substantial yield losses (up to 80% in severe epidemics), and they coexist in the same fields as tomato crops [6,9,10,11,12].

The interaction between *Fol* and tomatoes is one of the best-studied *F. oxysporum* pathosystems, and its impact on tomato production worldwide has been reduced due to the effective deployment of major disease resistance (R) genes. In tomatoes, four R genes (named *I* for immunity), *I*, *I-2*, *I-3*, and *I-7*, have been isolated [13,14,15,16]. The proteins encoded by the *I* genes can recognize specific effector proteins secreted by *Fol* (e.g., secreted in xylem (SIX) proteins) during host colonization [17]. The genes *SIX4* (*AVR1*), *SIX3* (*AVR2*), and *SIX1* (*AVR1*) are required for *I*-, *I-2*-, and *I-3*-mediated resistance to *Fol* races 1, 2, and 3, respectively [2,13,14,18,19]. Therefore, tomato-resistant materials carrying immunity genes (*I*, *I-2*, and *I-3*) have been developed and successfully deployed to control the three known races of *Fol* [17,20]. Moreover, *SIX* genes might be used to differentiate between *Fol* races and between *Fol* and other *F. oxysporum* ff. spp. [11,21].

Strains of *F. oxysporum* that infect different crop species have already been reported in Colombia. In the 1950s, *Fusarium oxysporum* f. sp. *cubense* (*Foc*) race 1 (i.e., the causal agent of Panama disease), was detected in the banana cultivar Gros Michael in the banana production region of Magdalena (Northern Colombia) [22]. Recently, Garcia-Bastidas et al. [23] reported a major outbreak of the highly aggressive f. sp. of *Foc* known as tropical race 4 (TR4), which struck banana plantations in Northern Colombia, threatening the entire banana production of the Americas. *Fusarium oxysporum* f. sp. *dianthi*, which infects carnations (i.e., the most limiting disease for this crop species), has been well characterized, with at least four pathogenic races identified, from which race 2 is the most frequent in this crop [24]. In another study, 12 isolates obtained from plant and animal tissues were identified as *Fusarium* spp., 7 of them grouped into the *F. oxysporum* species complex (FOSC), and were tested for their ability to infect different hosts (cross-infection) in plant hosts such as tomatoes, passionfruit, and carnations and in animal hosts. The isolates showed no cross-infection, suggesting host specificity for each f. sp. (e.g., *Fol* to tomatoes) [25].

In 2017, a total of 347,636 tons of tomato were produced in Colombia [26], representing 18.1% of national vegetable production. Despite the increase in production, there is a lack of knowledge about wilting diseases in the tomato cultivars used. Furthermore, outbreaks of vascular wilt disease (presumably caused by *F. oxysporum*) have been unofficially reported in tomato, cucumber, lentil, and chickpea crops from the Central Andean departments of Cundinamarca, Boyacá, and Valle del Cauca [22]. Currently, there is only one pathogenic isolate of *Fol* that has been identified by pathogenicity tests in 15 accessions of wild tomatoes [27]. However, information about the race of *Fol* or the presence of resistance genes in the plant accessions evaluated was not reported.

In Colombia, tomato production consists of the use of seeds of more than 60 different types of imported tomato hybrids, most of them with reported resistance to *Fol* races 1 and 2 [28]. Nevertheless, there is no information about the ff. spp. and physiological races affecting tomato crops. Therefore, the aim of this study was to perform an accurate identification of *Fol* strains from 120 *Fusarium* spp. isolates collected from wilted tomato plants in four different departments in the Central Colombian Andean Region (Cundinamarca, Boyacá, Antioquia, and Caldas) using a combination of morphological and molecular analyses. The morphological characterization was carried out by optical and scanning electron microscopy. The molecular analysis consisted of PCR screening of secretions in the xylem (*SIX*) effector genes, one new marker specific to *Forl* isolates and a phylogenetic analysis using the *translation elongation factor 1-alpha* (*EF1a*) gene of *Fusarium* species. Additionally, we performed pathogenicity assays to identify virulent strains of *F. oxysporum* on susceptible tomato plants. To the best of our knowledge, this is the first study describing the *Fol* races, *Forl*, and the nonpathogenic *F. oxysporum* isolates associated with the tomato cropping system in Colombia.

## 2. Results

### 2.1. Fungal Isolates from the Central Colombian Andean Region

**Figure 1:**
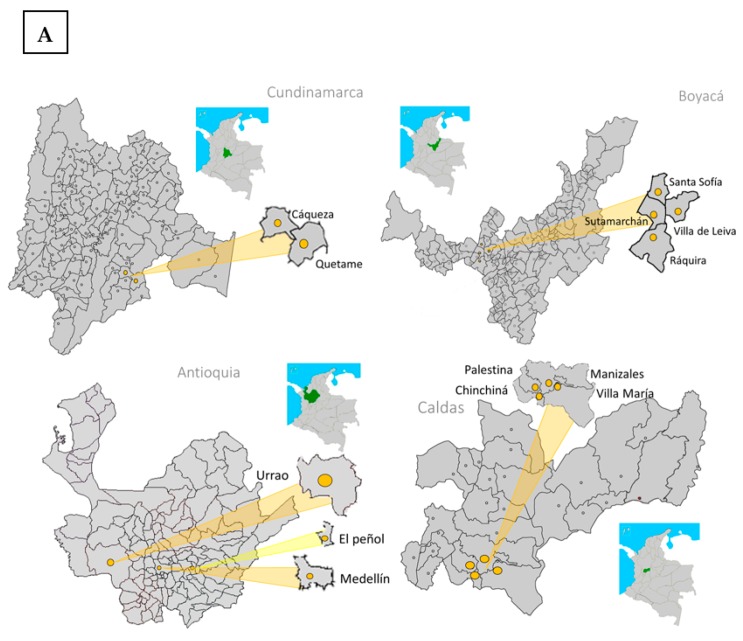

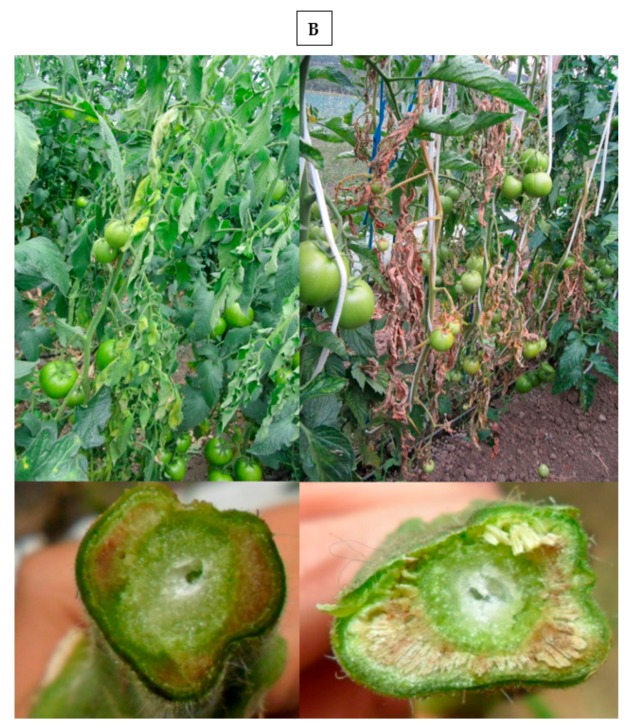
Location of the Central Colombian Andean Region, where 32 tomato fields with vascular wilting symptoms were visited. (**A**) Tomato fields visited in four different departments of the Central Colombian Andean Region (Cundinamarca, Boyacá, Antioquia, and Caldas). (**B**) Photographs of tomato plants showing symptoms of vascular wilt disease taken from the field locations visited in the Central Andean Region of Colombia. Upper part: plants showing wilting symptoms. Bottom: horizontal cortex of the stem, showing vascular browning of the xylem vessels. Pictures from Magda R. Gómez and Sandra L. Carmona. Photographic records from the Agricultural Microbiology Group, AGROSAVIA.

A total of 120 fungal isolates were obtained from 15 commercial hybrid tomato genotypes, collected from 32 tomato field locations that were visited in four departments of the Colombian Andean Region, including Antioquia, Boyacá, Caldas, and Cundinamarca (Table 1). Samples were collected from infected plants at different developmental stages between the first flowering (30 days old, approximately) and last fruit harvest (7 months old, approximately). The plants exhibited the typical symptoms of vascular wilt disease: wilting, chlorosis in the oldest leaves, and vascular browning, which is typical for *Fol* infections (Figure 1). Most of the isolates were obtained from four commercial hybrid tomato varieties: Chonto Aslam (16), Roble (14), Libertador (15), and Milano-Nicolas (19) (Table 1).

In 2017, a total of 32 tomato fields with vascular wilting symptoms and necrosis of the xylem vessels were visited in the departments of Cundinamarca, Boyacá, Antioquia, and Caldas, located in the Central Colombian Andean Region (Figure 1A,B). In those visits, a total of 222 growers were surveyed using two questions regarding *Fusarium* wilt disease and the types of chemicals applied to control the wilting disease symptoms observed. The questions were: (i) Has your tomato crop been affected by *Fusarium* wilt in the last year? (ii) Which chemical products do you use to control the disease?

The data collected revealed that 24% of the tomato crops visited were affected by a wilt disease (presumably caused by *Fusarium oxysporum*) in 2017. Moreover, in all of the tomato crops affected by the wilting disease, the farmers used chemical products to control the wilting symptoms observed. Farmers reported a total number of 49 different commercial products they used to control the wilt disease. Interestingly, only 14% of these commercial products have an active ingredient to control *Fusarium* spp., but they do not specifically control *Fol* or *Forl*.

### 2.2. Identification of F. oxysporum Isolates

#### 2.2.1. Morphology

Macroscopic analysis of the colony showed a variation in color between white, pale-to-dark violet, and magenta (Supplementary Materials, Figure S1A). Microscopic characteristics showed the formation of a slightly curved macroconidia over the sporodochia, with 3–4 septates and with a somewhat hooked apex and pedicellate base. The microconidia were oval, curved, or kidney-shaped, without or with one septate and formed over short monophialids and conforming pseudoheads. Microconidia were the most abundant structure in the culture media. All isolates also formed intercalary or terminal chlamydospores on hyphaes, which had a thickened cell wall. Microconidia ranged in size from 6.14 to 9.75 μm, macroconidia varied from 18.7 to 48.9 μm, and chlamydospores had a diameter from 7.5 to 8.4 μm (Supplementary Materials, Figure S1B). The fungal macroscopic characteristics and variations observed in the size and shape of the microconidia, macroconidia, and chlamydospores [29] suggested that the isolates collected may have corresponded to *Fusarium* spp.

#### 2.2.2. Molecular Identification of the Putative Fusarium Isolates

**Figure 2:**
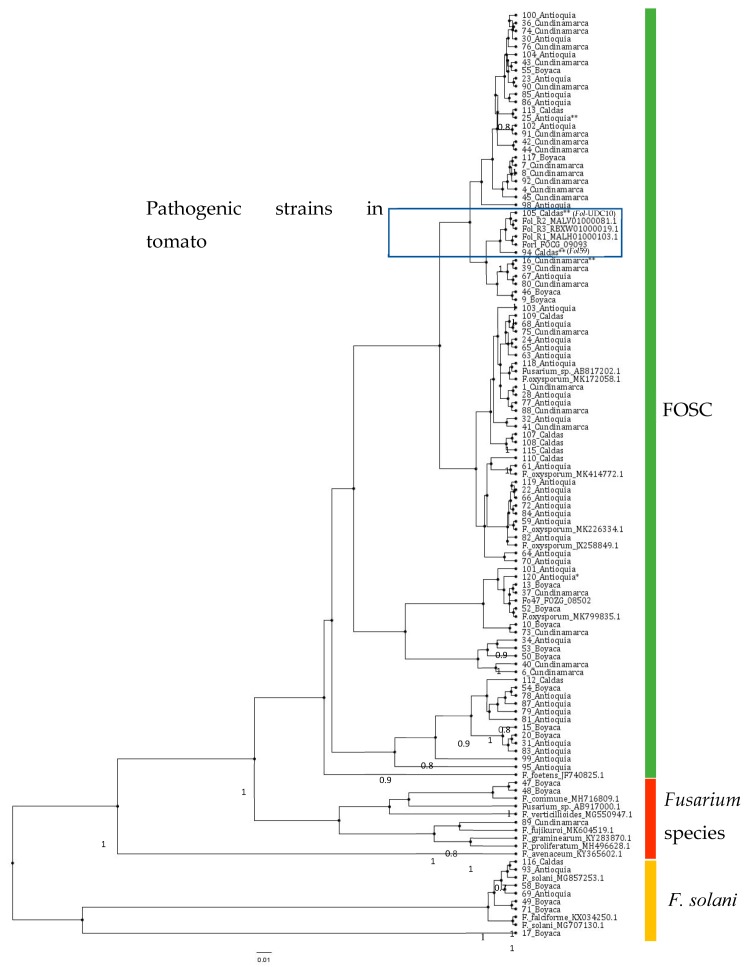
Phylogenetic tree of a partial sequence of the *EF1a* gene from 32 reference sequences of the *Fusarium* clade (Table 3) and the 95 fungal isolates obtained in this study. The node labels are the bootstrapping support, indicated as Bayesian probabilities. A key to the labels used for each taxa is provided in Table 1 and Table 3. The scale bar indicates time in millions of years. The pathogenic strains of *Fol* identified in this study (*Fol*59 and *Fol*-UDC10) are highlighted in dark blue to show their close phylogenetic relationship with reference strains of *F. oxysporum* f. sp. *radicis-lycopersici* (GenBank accession FOCG_09093) and *Fol* races 1, 2, and 3 (GenBank accessions MALH01000103.1, MALV01000081.1, and RBXW01000019.1, respectively). Strains with one asterisk indicate those positive for *Forl* molecular identification. Strains with two asterisks indicate ones used for pathogenicity tests. FOSC = *Fusarium oxysporum* species complex.

To further characterize the *Fusarium* isolates, we amplified the *Pg1* fragment (Table 2) in the 120 isolates obtained. In this PCR analysis, 105 isolates amplified a fragment that corresponded to the *Pg1* (Supplementary Materials, Figure S2). This result, together with morphology and microscopic analyses, suggested that 105 isolates out of the 120 collected might have been grouped into the *F. oxysporum* species complex. Moreover, in order to further support this evidence, we used the primers for the *EF1a* gene, designed by Cobo-Diaz et al. [30] (Table 2). The *EF1a* gene has been widely used for phylogenetic studies with isolates of the *Fusarium* genus [4,30,31]. In this study, 113 of the 120 isolates were amplified with *EF1a* (Supplementary Materials, Figure S3). This group of 113 isolates was used for further analyses with the *EF1a* gene. The PCR products of the *EF1a* obtained from the isolates was sequenced using the SANGER platform, and a total of 95 sequences were selected based on quality and length (≥350 bp), while 18 sequences were not considered for further analysis. A phylogenetic analysis was performed for the 95 fungal isolates with an *EF1a* sequence of good quality (Table 1). The analysis included 22 *EF1a* reference sequences of different *Fusarium* species and 10 *EF1a* reference sequences of *F. oxysporum* isolates, which were associated with tomato crops (Table 3, Figure 2). This analysis showed that the isolates were not grouped according to geographical location. Six of the ninety-five fungal isolates analyzed (49_Boyacá, 58_Boyacá, 69_Antioquia, 71_Boyacá, 93_Antioquia, and 116_Caldas) could be related to the *F. solani* clade, while three isolates (47_Boyacá, 48_Boyacá, and 89_Cundinamarca) were grouped close to *Fusarium* species different from *F. oxysporum*. The remaining fungal isolates were grouped with reference sequences of the *F. oxysporum* species complex (Figure 2).

#### 2.2.3. Identification of *Fusarium oxysporum* f. sp. *lycopersici* Candidate Isolates

**Figure 3:**
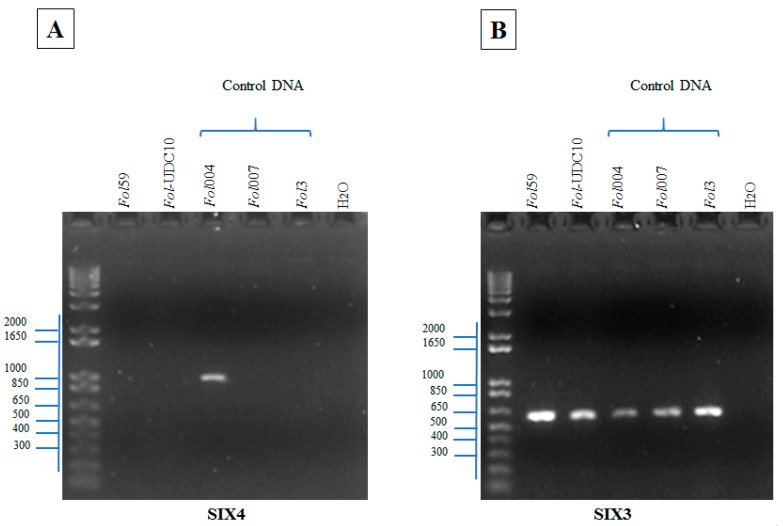
Polymerase chain reaction (PCR) analysis showing the presence of *Fusarium oxysporum* f. sp. *lycopersici* (*Fol*) *SIX4* and *SIX3* on the positive isolates and the presence of *SIX1* (Supplementary Materials, Figure S4A). (**A**) PCR amplification of *SIX4*, showing a band of the expected size (967 bp) only in the DNA from the strain *Fol*004, which was used as a positive control for *Fol* race 1. (**B**) PCR amplification of *SIX3*, showing a band of the expected size (608 bp) in the DNA of the Colombian isolates *Fol*59 and *Fol*-UDC10, compared to the control DNA from the three races. Colombian isolates *Fol*59 = 94 and *Fol*-UDC10 = 105 (Table 1, Supplementary Materials, Figure S4). Control DNA: *Fol*004 = race 1, *Fol*007 = race 2, and *Fol*3 = race 3; bp: base pair. A fragment of approximately 608 and 967 bp was expected for *SIX3* and *SIX4*, respectively. PCR product visualization was carried out following electrophoresis in a 1% agarose gel.

**Figure 4:**
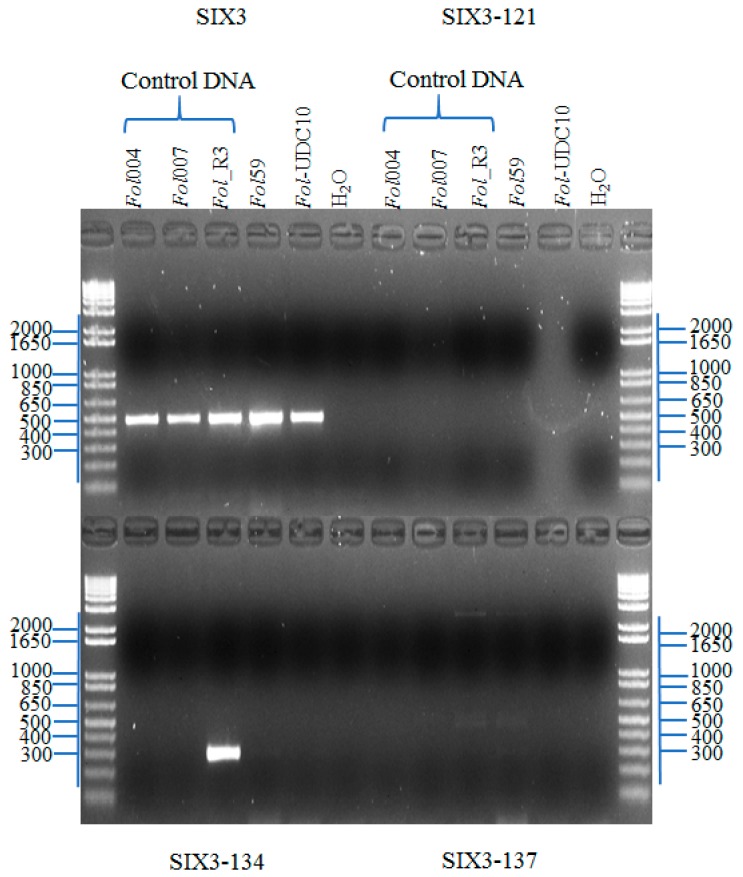
Polymerase chain reaction (PCR) analysis showing a comparison between the presence of *Fol SIX3* gene and the possible Single Nucleotide Polymorphisms (SNPs) on the coding sequence (CDS) of five isolates of *Fol.* It has been already reported that *Fol* race 3 isolates differing in a single nucleotide from race 1 and race 2 isolates (within the CDS: G121 > A, G134 > A and G137 > C [32]). The upper left part of the gel shows the bands corresponding to the *SIX3* CDS using SIX3-F1/SIX3-R2 primers. Based on these three SNPs differences on *SIX3* gene, three primer pairs were developed by Lievens et al. [32] (SIX3-G121A-F2/SIX3-R2, SIX3-G134A-F2/SIX3-R2 and SIX3-G137C-F1/SIX3-R2) enabling unambiguous PCR differentiation of race 2 and race 3 isolates when using stringent PCR conditions. The lower left part of the figure shows an amplification of the *SIX3* gene using primers SIX3-G134A-F2/SIX3-R2 in the *Fol*_R3 control strain for *Fol* race 3, indicating that *Fol*_R3 carry the point mutation G134 > A on *SIX3* gene. The right part shows no PCR products using primers SIX3-G121A-F2/SIX3-R2 or SIX3-G137C-F1/SIX3-R2 in any of the five strains analyzed. *Fol*004 = *Fol* race 1, *Fol*007 = *Fol* race 2, and *Fol_R*3 = *Fol* race 3 (control strains). *Fol*59 and *Fol-*UDC10 = the Colombian isolates analyzed in this study. A fragment of approximately 429, 414, or 412 bp was generated when using SIX3-G121A-F2/SIX3-R2, SIX3-G134A-F2/SIX3-R2, or SIX3-G137C-F1/SIX3-R2, respectively. PCR product visualization was carried out following electrophoresis in a 1% agarose gel.

**Figure 5:**
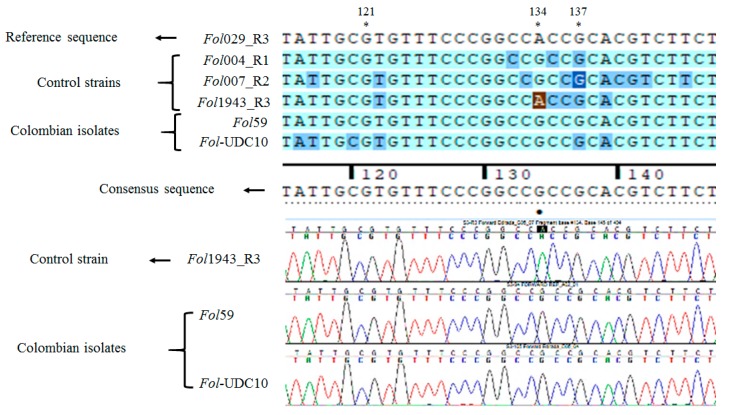
Schematic representation of the nucleotide sequence alignment of the *SIX3* gene, comparing the sequences of five different isolates. The brown highlighted nucleotide in the alignment and the bullet point in the chromatograms indicate the presence of an SNP, A/G, in nucleotide 134, which was only observed in the sequencing products of the *Fol*1943 and *Fol*029 strains, which were used as a control and reference sequence of *Fol* race 3, respectively. No SNPs were identified in the rest of the sequencing products of the *SIX3* gene from *Fol* races 1 and 2 and the Colombian isolates. *Fol*029_R3 = the reference sequence for *SIX3* used in this analysis. Variable nucleotides are indicated with *. *Fol* 004_R1, *Fol*007_R2, and *Fol*1943_R3 = the sequencing products of *SIX3* used as a control for *Fol* races 1, 2, and 3, respectively. *Fol_*59 and *Fol*_UDC10 = the sequencing products of the *SIX3* gene from the *Fol* Colombian isolates. The alignment and SNP identification analyses were performed with the software Sequencher^TM^ v4.9.

To perform a rapid and unambiguous identification of *Fol* from the fungal isolates described before, we used the effector genes *SIX1*, *SIX3*, and *SIX4*, which are specific pathogenic strains of the *F*. *oxysporum* species complex [18,21,35], as molecular markers. The effector *SIX1* is present in the majority of pathogenic strains of *Fol* [21]. In this study, *SIX1* was identified in 2 isolates (94 and 105, named hereafter as *Fol*59 and *Fol-*UDC10, respectively) out of the 120 fungal isolates (Table 1, Supplementary Materials, Figure S4A). In *Fol*, the effector gene *SIX4* was only present in strains classified as race 1 [36]. Thus, we used *SIX4* to determine whether *Fol*59 and/or *Fol-*UDC10 could correspond to race 1. However, no amplification of *SIX4* was observed in either *Fol*59 or *Fol-*UDC10 (Figure 3A, Supplementary Materials, Figure S4B). To determine whether *Fol*59 and *Fol-*UDC10 might correspond to *Fol* race 2 (lost *SIX4*, carrying *SIX1* and *SIX3*) or race 3 (lost *SIX4*, carrying *SIX1* and *SIX3* with Single Nucleotide Polymorphisms (SNPs) in the nucleotides 121, 134, or 137) [21], a PCR amplification and a sequencing of *SIX3* were performed on both *Fol* candidate isolates (Figure 3B and Figure 4). *SIX3* was identified on *Fol*59 and *Fol-*UDC10 (Supplementary Materials, Figure S4C) isolates, and the sequencing analysis of the *SIX3* Coding Sequence (CDS) suggested that both *Fol*59 and *Fol-*UDC10 belonged to *Fol* race 2, as no polymorphism was found in *SIX3* (compared to the corresponding reference sequences of *Fol* races 2 and 3) (Figure 5).

#### 2.2.4. Identification of *Forl* Candidates

**Figure 6:**
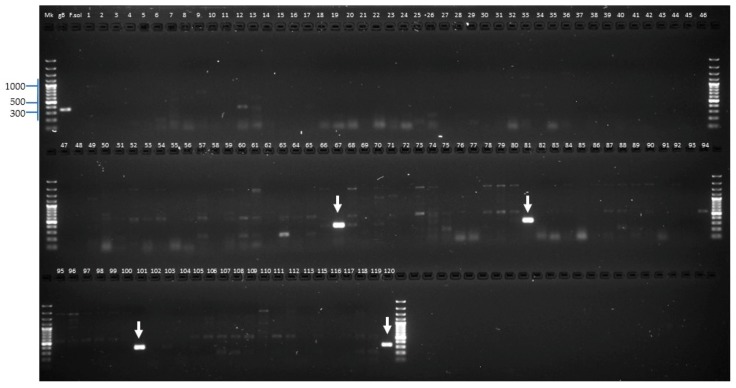
PCR analysis showing the amplification of a new specific marker (*Forl*_155.3) developed for *Forl* in this study. The four positive bands are indicated by white arrows (expected size 337 bp). A DNA fragment contained in the *Forl*_155.3 region was synthesized (gBlock, Integrated DNA Technologies, Coralville, IA, USA) and used as a positive control (gB). The DNA of *Fusarium solani* was used as a negative control (F.sol). Mk: Gene Ruler 100 bp Plus DNA Ladder, Thermo Scientific Waltham, MA, USA. PCR product visualization was carried out following electrophoresis in a 1% agarose gel.

A specific primer pair that could distinguish *Forl* from *Fol* was designed. Briefly, the genome sequences of *Fol* race 2 (GCA_000259975.2), *Fol* race 3 (GCA_000149955.2), and *Forl* (GCA_000260155.3) were compared, and a pair of primers (*Forl*_155.3) specific to a region of 337 bp that is only present in the *Forl* genome was designed. We used the *Forl*_155.3 primers in the 118 fungal isolates (with no evidence of the presence of *SIX* genes) and both *Fol*59 and *Fol*UDC-10 as a negative control. As a result, the newly designed marker specific for *Forl* isolates was amplified only in 4 isolates (Fut52, Fur55, Fur38, and Fut64) out of the 120 tested (Table 1 and Figure 6). As shown in Figure 6, a specific and well-defined PCR band was observed for the *Forl* positive isolates.

### 2.3. The Colombian Isolates Fol59 and Fol-UDC10 Were Highly Virulent on Susceptible Tomato Plants

**Figure 7:**
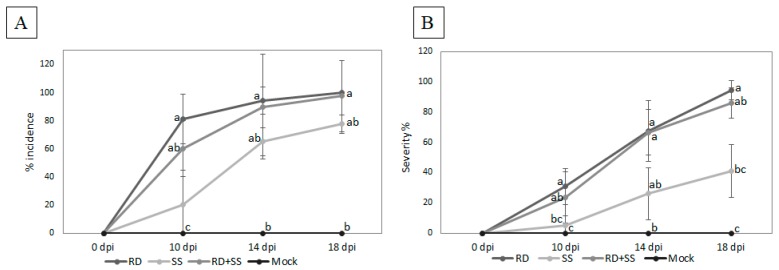
Inoculation methods tested in this study. Disease incidence and severity were evaluated at four different time points (0, 10, 14, and 18 days post-inoculation) with *Fol-*UDC10. The percentage of disease incidence (**A**) and severity (**B**) were significantly higher in plants inoculated with *Fol*-UDC10 using the root-dipping method compared to those inoculated using the soil-suspension method. RD = root-dipping inoculation method, SS = soil-suspension inoculation method, RD + SS = mix of root-dipping and soil-suspension inoculation methods. Treatments with different letters are significantly different at *p* = 0.05.

A comparative analysis of the three inoculation methods (soil suspension (SS), root dipping (RD), and an RD + SS combination) was performed in tomato seedlings two, three, and four weeks old of the cultivar Santa Cruz Kada (susceptible to all three races of *Fol*) with the Colombian isolate *Fol-*UDC10. Plants inoculated using both the RD and RD + SS methods exhibited typical symptoms of vascular wilt disease (e.g., chlorosis of the lower leaves and wilting) (Supplementary Materials, Figure S5). After 18 dpi, the plants inoculated using both the RD and RD + SS methods showed significantly higher incidence and severity of wilting disease symptoms compared to those inoculated using the SS method (Figure 7). Although there were no significant differences between RD and RD + SS, the RD method was selected for further analysis (using both *Fol*59 and *Fol-*UDC10 isolates) due to the development of the typical symptoms of *Fusarium* wilt (Supplementary Materials, Figure S5).

Once the RD infection protocol was selected, seedlings of three different ages (i.e., two, three, and four weeks old) were inoculated with *Fol*59 in order to describe the typical wilting symptoms appropriately and to describe the disease progress in a time course experiment. After 14 dpi, plants corresponding to all three ages showed the typical disease symptoms of *Fusarium* wilt, with an incidence ranging from 95% to 100%. Disease incidence and severity were not significantly different when comparing inoculated plants at different ages. Nevertheless, both disease symptoms and progress were better recorded using four-week-old plants (Supplementary Materials, Figure S5). Therefore, four-week-old seedlings of Santa Cruz Kada were used in further pathogenicity tests.

**Figure 8:**
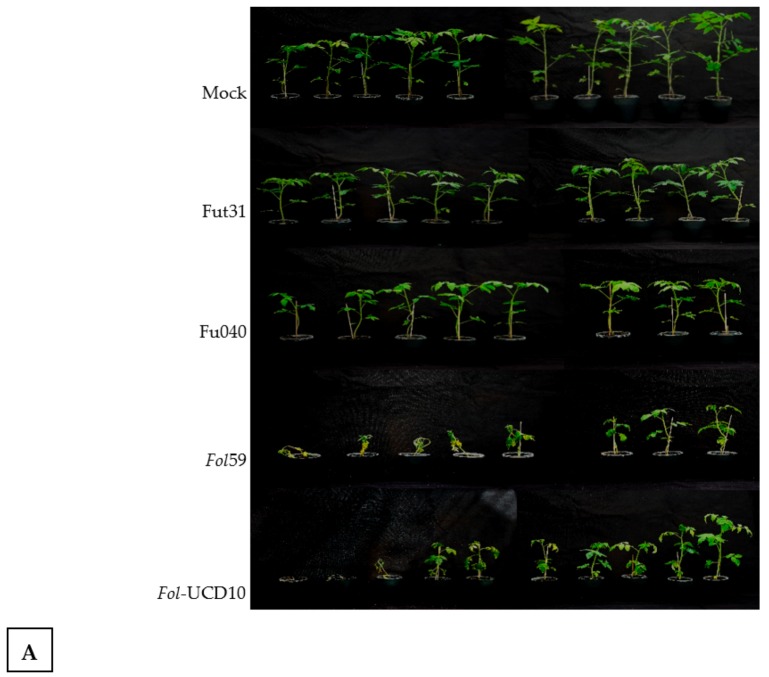

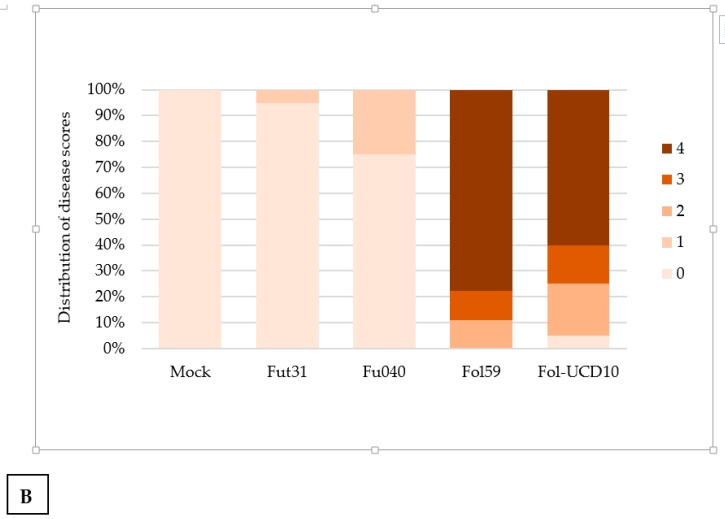
Pathogenicity tests on Santa Cruz Kada tomato plants with the *F. oxysporum* isolates Fut31, Fu040 (reference strain), *Fol*59, and *Fol*-UDC10. (**A**) Photographs taken at 21 days post-inoculation (dpi) of infected Santa Cruz Kada plants from one experiment. (**B**) Distribution of disease scores for plants shown in (**A**), pooled from two replicate experiments (*n* = 18–20). Probability values were obtained using the nonparametric Mann–Whitney test to determine significant differences in disease scores between plants infected with different fungal isolates. Treatments with different letters are significantly different at *p* = 0.05. Photographs taken by Jaime Simbaqueba. Photographic records are from the Agricultural Microbiology Group, AGROSAVIA.

In further plant disease assays, the RD inoculation method was carried out with a spore suspension of 5 × 10^6^ conidia mL^−1^ using the preselected isolates *Fol*59 and *Fol-*UDC10. To test whether *Fol*59 and *Fol-*UDC10 isolates were indeed virulent, four-week-old seedlings were inoculated with both isolates, and severe disease symptoms were observed after 21 dpi (Figure 8A,B). Non-inoculated plants (mock control) as well as *Fol* isolates Fut31 and Fu040 (which lack the *SIX* genes and were identified as *F. oxysporum* in this study, but with no evidence that they are pathogenic in tomato-susceptible plants) served as controls. The distribution of disease scores for susceptible plants to *Fol* (Figure 8B) showed that plants inoculated with Fut31 and Fu040 presented mostly a score of 0 (indicating healthy plants), whereas *Fol*59- and *Fol-*UDC10-inoculated plants were scored as 4, indicating wilted plants with brown coloration in vascular bundles. This result confirmed that *Fol*59 and *Fol-*UDC10 were highly virulent strains of *Fol*.

### 2.4. Pathogenicity Assays with the Forl Candidate Isolates Showed Root Rot Symptoms in Tomato Plants

In the tomato plants with *Fusarium* wilt symptoms sampled in the Central Andean departments of Colombia, four isolates (Fut52, Fur55, Fur38, and Fut64) were identified as *Forl* using the new diagnostic molecular marker (*Forl*_155.3) reported in this study (Table 1 and Figure 6). To investigate whether these isolates identified as *Forl* could produce *Fusarium* crown and root rot (FCRR) symptoms, tomato plants 30 days old were inoculated through root dipping using a microconidia suspension of *Forl*, and plants non-inoculated with *Forl* were used as a control. After 60 dpi, external symptoms of FCRR, including brown discoloration and rot in the crown and root, were observed (Supplementary Materials, Figure S6).

## 3. Discussion and Conclusions

In Colombia, a total of 347,636 tons of tomato were produced during 2017, representing one of the most cultivable vegetable crops in the country. However, this crop is highly susceptible to biotic and abiotic stresses, which result in an increase in production costs [26,28]. *Fusarium* wilt is currently a disease of major significance throughout the country. Varietal resistance is a highly efficient alternative to control the disease, using a number of commercial tomato hybrid varieties derived from the introgression of resistance genes specific to *Fol* races 1 and 2 [11]. The seeds of tomato hybrids used in Colombia are imported, and there is no information about *Fol* races present in the country. Tomato producers located in departments with high yields, such as Cundinamarca, Boyacá, Antioquia, and Caldas, use tomato seeds with no information about resistance to local populations of *Fol* [28]. The lack of knowledge about the causal agent of the vascular wilting symptoms affecting tomato crops makes the development of adequate management practices difficult. After varietal resistance, the use of chemical fungicides has been the most common management strategy for disease symptoms caused by either *Fol* or *Forl* [11]. The survey carried out in this study indicated that Colombian farmers use agrochemicals to control *Fusarium* wilt, in most cases without knowledge. However, there is no suitable fungicide for controlling the wilt disease caused by *Fol* [3,6]. The average use of chemical pesticides for the year 2015 in Colombia was 14.7 kg/ha, and Colombia was third in the word in terms of pesticide consumption (compared to a 3.9 kg/ha worldwide average). The control of *Fusarium* wilt should be based on an integrated disease management (IDM) strategy based on monitoring, economic thresholds, and preventive approaches to determine when and how fungicide application is needed [37]. Despite the fact that appropriate control strategies for wilt disease that could be implemented in Colombian tomato crops exist (reviewed in Reference [38]), a suitable IDM strategy in Colombia to control *Fusarium* wilt in tomatoes has not been established due to the absence of knowledge, sources, and socioeconomic conditions among producers.

This study presents the current state of pathogenic and nonpathogenic *F. oxysporum* populations associated with tomato fields in four different regions from the Central Colombian Andean Region. Most of the fungal isolates obtained exhibited a variable range of morphological characteristics of *F. oxysporum* (e.g., colony color, texture, and sporulation) according to the taxonomic description reported in References [29,39]. Combining all of the analyses performed using the new specific markers for the *Forl*_155.3, *SIX*, and *EF1a* genes in the 120 isolates obtained in this study, 2 isolates were identified as *Fol* (*Fol*59 and *Fol*-UDC10), 4 isolates were identified as *Forl* (Fur38, Fut64, Fut52, and Fur55, Table 1), 5 isolates belonged to the *F. solani* clade (49_Boyacá, 58_Boyacá, 69_Antioquia, 71_Boyacá, 93_Antioquia, and 116_Caldas, Table 1), 3 isolates clustered were with other *Fusarium* species different from *Fol* and *Forl* (47_Boyacá, 48_Boyacá, and 89_Cundinamarca, Table 1), and finally, most of the fungal isolates were clustered with reference sequences of the *F. oxysporum* species complex and could potentially correspond to nonpathogenic strains of *F. oxysporum*.

To test the potential of *SIX* genes as pathogenicity markers, we initially assessed the presence of *SIX1*, *SIX3*, and *SIX4* in a collection of 120 *F. oxysporum* isolates. Only two isolates, *Fol*59 and *Fol-*UDC10, were positive for *SIX1* and *SIX3* amplification. Then, the absence of *SIX4* and SNPs in the coding sequence of *SIX3* on *Fol*59 and *Fol-*UDC10 (compared to the corresponding reference sequences in *Fol* races 1, 2, and 3) indicated that these two isolates belonged to *Fol* race 2 [11,21]. Pathogenicity assays showed that *Fol59* and *Fol-*UDC10 were both highly virulent in tomato-susceptible plants compared to the isolate Fut31 (25_Antioquia, Table 1) obtained in this study and to the local reference strain Fu040 (16_Cundinamarca, Table 1) of *Fusarium oxysporum*. As a result, no disease symptoms were observed in plants inoculated with Fu040, contrary to the previous evidence described by Moreno et al. [40]; however, different inoculation methods, experimental conditions, and plant material were used compared to the results obtained by Moreno et al. [40].

In addition, the *SIX* genes evaluated were found to be absent in the other 118 nonpathogenic isolates (including Fut31 and Fu040), thus showing a direct correlation between the presence of *SIX* genes and the ability of *Fol* isolates to cause severe wilting symptoms (Figure 7). Previous studies have demonstrated the usefulness of *SIX* genes for the discrimination of *Fol* races [7,21]. These results propose a basis for an understanding of the races of *Fol* present in Colombia and for establishing a linkage between races and commercial tomato hybrid varieties. As mentioned before, more than 90% of the commercial tomato seeds growing in Colombia are introduced from other countries without adequate resistance tests for local *Fol* and *Forl* populations, and there is deficient technological appropriation in terms of the conditions of specific microenvironments.

During the isolation process of the causal agent of the wilting symptoms from infected plant tissues, it is common to recover more than one species of *Fusarium* (as they are common inhabitants of soil and the rhizosphere), but some species or strains may be pathogens, while others are saprophytes or endophytes and have no role in the disease process whatsoever [29,41,42,43].

A phylogenetic analysis of the 95 isolates using the *EF1a* gene showed that 7 of them could be related to other *Fusarium* species (*F. solani*, *F. falciforme*, and *F. fujikuroi*), while 82 corresponded to *F. oxysporum* (possibly nonpathogenic isolates due to the absence of any of the *SIX* effector genes analyzed). This evidence agrees with other studies where *F. oxysporum* nonpathogenic isolates were collected from infected plant tissues from tomato crops in India and Algeria and from soil samples from Florida (the United States) [41,42,43].

Therefore, nonpathogenic strains of *F. oxysporum* are commonly isolated from tissue samples of wilted tomato plants. The *F. oxysporum* species complex also includes numerous nonpathogenic strains, some of which have been shown to be effective in plant protection or biocontrol [44,45]. According to our results, we hypothesize that when the tissue samples were collected to isolate pathogenic strains of *F. oxysporum*, there was a high proportion of nonpathogenic *F. oxysporum* strains, and we obtained more nonpathogenic isolates during the purification of the fungal cultures. The nonpathogenic isolates collected in this work may therefore represent interesting candidates for further analyses on alternative biocontrol approaches to control *Fusarium* wilt.

It is important to note that after the phylogenetic analysis of *EF1a* sequences, *Forl* isolates were not clustered alone or with any *Fusarium* pathogenic strains in tomatoes. They were scattered within the FOSC group, without evidence of a phylogenetic structure with respect to pathogenicity (Figure 2). This suggests that *EF1a* might not be useful in resolving the phylogenetic positions of the *Forl* isolates studied. Thus, further studies are needed using the polymorphisms in DNA sequences for other markers, such as RNA polymerase II (*rpb2*) or the Internal Transcribed Spacer (ITS) region, to gain insight into the phylogenetic relationship between *Fol* and *Forl* within the FOSC group.

In addition, the results obtained in this study have another practical consequence for Colombian tomato crops: the *SIX* genes and the specific marker *Forl*_155.3 may be used as specific markers in epidemiological studies of *F. oxysporum* pathogenic isolates, for rapid diagnosis, and for discrimination from nonpathogenic isolates. Such tools, which are applicable for both diagnosis and tracking, will help to prevent the spread of pathogenic strains of *Fol* and *Forl* to other regions in Colombia and to nearby countries.

Knowledge of the *Fol* physiological races affecting tomato crops will be useful for the development of better disease management strategies, including plant breeding and tomato grafting. Part of the diversity of tomato genetic resources (more than 1000 accessions) is maintained in the Tomato Germplasm Bank (TGB) located in the Colombian Agricultural Research Corporation (AGROSAVIA). The majority of accessions contained in the TGB have not yet been characterized for their resistance/susceptibility to any phytopathogen, such as *F. oxysporum*. Therefore, this study provides primary insight into the search for tomato-resistant genotypes and their interactions with local *Fol* and *Forl* populations and thus contributes to improving management strategies for this important disease.

## 4. Materials and Methods

### 4.1. Fusarium Isolates and Culture Conditions

Samples consisting of roots and stem tissues from wilted tomato plants (Table 1) were collected from the surveyed fields, wrapped in plastic bags, and carried to the Agricultural Microbiology Laboratory at the Tibaitatá Research Center of AGROSAVIA-Colombia for subsequent analysis. The samples were surface-sterilized using 2% sodium hypochlorite (NaOCl) solution for 15 min and 70% ethanol for 1 min, and they were finally rinsed three times in sterile water. For each sample, plant tissue was dried, sectioned in six segments of 0.5 cm, and placed directly on one plate of Potato Dextrose Agar (PDA; Merck, Darmstadt, Germany) amended with 0.1% Triton 100X (Sigma-Aldrich^TM^, Saint Louis, MO, USA) and 0.3% chloramphenicol to avoid bacterial contamination. Plates were incubated at 25 ± 2 °C in 24-h light conditions for four days. The plates were observed routinely, and all colonies with typical *Fusarium* morphology were checked using optical microscopy for microconidia, macroconidia, and chlamydospores. Then, positive colonies were transferred to fresh PDA until pure fungal colonies were obtained, from which monosporic cultures were obtained. Two long-term fungal culture preservation methods were used: filter paper and 30% (*v*/*v*) glycerol–water.

### 4.2. Morphological Characterization

Fungal isolates were plated onto Carnation Leaf Agar (CLA) medium [39] and incubated at 25 °C for three to five days. Propagative structures of *F. oxysporum* were observed using an optical microscope (Olympus CX31™, Tokyo, Japan). To perform environmental scanning electron microscopy, samples collected from fields with vascular wilting symptoms were disinfected with a 1% NaOCl solution for 1 min and 70% ethanol for 50 s, triple-washed with distilled sterile water, and transferred onto a sterile paper towel to remove excess moisture. A portion of the infected material was placed on PDA medium and incubated for 5 days at 28 °C until pure fungal colonies were obtained. Visualization of the samples was carried out using a scanning electron microscope (ESEM-FEI QUANTA 250, FEI Co., Hillsboro, OR, USA) located in the Instituto de Estatigrafía de la Universidad de Caldas, Colombia. An untreated mycelium portion was cut, fixed, and placed on aluminum stubs (FEI Co., Hillsboro, OR, USA) double-coated with conductive carbon tape. The samples were examined with the ESEM using a secondary electron detector (SE), an acceleration voltage of 10 kV, a working distance of 10 mm, and a magnification of 5000×. The morphological and microscopic characteristics were described according to Leslie and Summerell [29].

### 4.3. DNA Extraction and PCR Analysis

The purified isolates were grown on PDA, and DNA was extracted from 100 mg of the mycelia from each isolate using the cetyl trimethylammonium bromide (CTAB) protocol [46] modified for fungal DNA. Additionally, DNA from the reference strains *Fol*004 (race 1), *Fol*007 (race 2), and *Fol*1943 (race 3), kindly provided by Professor David Jones of the Australian National University, were used as a positive control. The sets of primers Uni-F and Uni-R, which amplify a fragment of 670~672 bp (from the endo-polygalacturonase gene (*Pg1*) [32]), and EF1-F and EF1-R, which amplify a fragment of approximately 770 bp (from the *translation elongation factor 1-alpha* (*EF1a*) gene of *F. oxysporum* (GenBank: MK172058.1), reported by Cobo-Diaz et al. [30] (Table 2)), were used in order to determine whether the isolates could correspond to the *F. oxysporum* species complex. An identification of the effector gene *SIX1* (using the primers P12-F and P12-R (Table 2)) was performed to determine putative *Fol* isolates. Additionally, primers that amplify the effector genes *SIX4* and *SIX3* (Table 2) were used to determine the race of the *Fol* isolates (i.e., isolates positive for *SIX4* were classified as race 1, while negatives for *SIX4* but positives for *SIX3* could be classified as either race 2 or 3) [18,21,35,36]. The primer pairs SIX3-F1/SIX3-R2, SIX3-G121A-F2/SIX3-R2, SIX3-G134A-F2/SIX3-R2, and SIX3-G137C-F1/SIX3-R2 were used to differentiate between *Fol* races 2 and 3 [21]. DNA from the reference strains *Fol*067 (*Fol*MM10), *Fol*029 (5397), and *Fol*035 (IPO3), which belong to *Fol* race 3 and contain SNPs on the nucleotides 121, 134, and 137 of *SIX3*, respectively, were kindly provided by Dr. Martijn Rep, University of Amsterdam, and were used as a control.

To identify putative *Forl* isolates, a specific pair of primers (named *Forl*_155.3) was designed based on a comparison of the genome sequences of *Fol* race 2 (NCBI genome accession GCA_000259975.2), *Fol* race 3 (GCA_000149955.2), and *Forl* (GCA_000260155.3). A DNA fragment that contained the *Forl*_155.3 region was synthesized (gBlock, Integrated DNA Technologies, Coralville IA, USA) and used as a positive control.

For amplification of the *SIX* genes and the *Forl*_155.3 fragment, PCR reactions were conducted with Taq DNA Polymerase (Invitrogen™, Carlsbad, CA, USA) in a 25-µL reaction volume. The PCR reaction consisted of 0.25 µL Taq Polymerase, 2.5 µL of 10X buffer (Invitrogen™, Carlsbad, CA, USA), 0.16 µM of each primer, 0.16 mM of dNTP mix, 2 mM MgCl_2_, and 25 ng of template DNA. PCRs were carried out with an initial denaturing step at 95 °C for 2 min, followed by 30 cycles of denaturing at 95 °C for 45 s, the annealing of primers at 59 °C (62 °C for *Forl*_155.3) for 45 s, and primer extension at 72 °C for 45 s. The PCR was completed by a final extension at 72 °C for 10 min. The melting temperature for each primer was determined using a temperature gradient. A touchdown PCR [47] was performed to amplify the primer pairs for the *SIX3* gene as follows: the first phase consisted of 10 cycles with an annealing temperature of 69 °C for the first PCR cycle, which decreased by 1 °C per cycle until an optimal annealing temperature of 59 °C was reached. The remaining cycling conditions consisted of 20 cycles with an annealing temperature of 59 °C. Amplification was visualized on 1.4% *w*/*v* agarose gel stained using SYBR™ Safe DNA Gel Stain (Invitrogen™, Carlsbad, CA, USA).

### 4.4. Sequencing Analysis of the EF1a Gene among the Fungal Isolates

The *EF1a* gene was amplified by PCR with the same conditions used for the *SIX* genes (described above) with modifications of the annealing temperature at 62 °C. The PCR products of the *EF1a* gene, which were obtained from the 120 fungal isolates, were sequenced by SANGER (Applied Biosystems/Thermo Fisher Scientific, model ABI 3500 Genetic Analyser, Foster City, CA, USA), trimmed for sequence quality using the software Geneious v.2019.2.1 [48], and compared to 12 *EF1a* reference sequences reported for other *Fusarium* species and 10 *EF1a* sequences reported for *F. oxysporum* isolates associated with tomatoes (Table 3) (using the software MAFFT) [49,50]. Phylogenetic analysis was performed with the multiple sequence alignment of the *EF1a* gene mentioned above using the software BEAST (Bayesian Evolutionary Analysis Sampling Trees) v2.6.1 [51,52] with default settings. The resulting phylogenetic trees were visualized using Figtree v1.4.3 [53].

Sequences of *SIX1*, *SIX3*, and *EF1a* from the fungal isolates obtained in this study were deposited in the GenBank database under accession numbers MN745203, MN745204, MN745207, MN745208 (*SIX1* and *SIX3*), and MN745105-MN745199 (*EF1a*). Unique sequence identifiers and annotation information are provided in the Supplementary Materials, Table S1.

### 4.5. Pathogenicity Assays

Three inoculation methods were evaluated using the *Fol*59 isolate with the presence of *SIX* genes on the susceptible tomato cultivar Santa Cruz Kada (Impulsemillas™, Bogotá, Colombia) in order to establish a reliable pathogenicity assay with a virulent strain of *Fol* and susceptible tomato plants. Seeds were disinfected using 2% NaOCl solution for 10 min and then 70% ethanol for 1 min, rinsed three times in sterile water, and finally sown in sterile peat. Fifteen-day-old seedlings were inoculated with *Fol* microconidia using three methods, as follows: (i) Soil suspension (SS) consisted of transplanting the seedlings to 0.5-L pots with a sterile vermiculite/soil (2:8) mixture containing 1 × 10^6^ microconidia mL^−1^ of *Fol* suspension. Seedlings transplanted to pots with a sterile mixture and mock-inoculated plants with water were used as a control. (ii) Root dipping (RD), the second method, which has been described by Mes et al. [54] and adapted by Rep et al. [55], Gonzalez-Cendales et al., [15] and Simbaqueba et al. [56], with modifications, consisted of the uprooting of the seedlings, preserving the root integrity. The roots were submerged for 15 min in a 5 × 10^6^ microconidia mL^−1^ suspension of *Fol*. Seedlings dipped in sterile water served as a mock for inoculation. (iii) The third method was a combination of the first two methods mentioned. Additionally, seedlings from three developmental times (i.e., 15 days old, 25 days old, and 30 days old, or the seedling stage for transplanting) were tested using the most effective inoculation method. The plants were maintained in a controlled-environment growth room with a 12 h/30 °C day (100 mmol m^−2^ s^−1^) and a 12 h/30 °C night cycle for the first 6 days post-inoculation (dpi). They were transferred to greenhouse conditions until 14 to 18 dpi. Plants were sampled according to external disease symptoms every four days during the infection process.

The pathogenicity assays of *Fol* consisted of a randomized complete-block design, where four different treatments were compared: SS, RD, RD + SS, and mock-inoculated plants. To maximize the statistical reliability of the data, three biological replicates were carried out, and for each biological replicate, four technical replicates were performed, where the experimental unit consisted of 5 plants. The number of wilted leaves per plant was recorded as a *Fol* symptom, according to the disease severity scale reported by Cakir et al. [57], Akhter et al. [58], and Rongai et al. [59] and modified for this study (Supplementary Materials, Table S2 and Figure S5). The severity disease index was calculated using the Equation (1) described by Chiang et al. [60]:

(1) $$DSI(\%)=\frac{\sum \left(disease\;class\;frequency\times disease\;class\right)}{\left(\#\;total\;observations\right)\times \left(highest\;disease\;level\right)}\times 100$$

Differences in the disease incidence and disease severity between treatments were tested for statistical significance with ANOVA and Kruskal–Wallis tests using the software Statistix version 8.0 with a probability value of *p* = 0.05. Additional plant wilting symptoms and vascular browning were recorded and used to calculate disease scores according to the following criteria (described by Rep et al. [61], Gonzalez-Cendales et al. [15], and Simbaqueba et al. [56]): 0, healthy plant; 1, slightly swollen or bent hypocotyl; 2, one or two brown vascular bundles in the hypocotyl; 3, at least two brown vascular bundles and growth distortion; and 4, all vascular bundles brown and plant either dead or very small and wilted. Differences in the distributions of disease scores between treatments were tested for statistical significance through pairwise two-tailed Mann–Whitney tests [62].

For the pathogenicity test with *Forl* isolates, we performed the same inoculation procedure used for *Fol* with modifications in the controlled-environment growth room (with a 12 h/25 °C day cycle and a 12 h/25 °C night cycle for 60 dpi). Plants were considered diseased when they exhibited brown necrosis on the crown and vascular discoloration in the lower stem (Supplementary Materials, Figure S6).

## Figures and Tables

**Table 1:** Endophyte fungal isolates found in 32 tomato fields in the Colombian Andean Region.

Department	Field Location	Coordinates		Tomato Cultivar	*Fol* Resistance	Isolates and Tissue of Sample Collection	
	Town	Latitude	Longitude			Isolates from Stem	Isolates from Roots
Cundinamarca	Cáqueza	N.A.	N.A.	Libertador ^a^	R1, R2		36_Cundinamarca
Cundinamarca	Cáqueza	N.A.	N.A.	Nicolas ^b^	R1, R2		37_Cundinamarca
Cundinamarca	Cáqueza	4°39′84.46″	73°94′90.32″	Nicolas ^b^	R1, R2	1_Cundinamarca	38_Cundinamarca
Cundinamarca	Cáqueza	N.A.	N.A.	Libertador ^a^	R1, R2	2_Cundinamarca	39_Cundinamarca
Cundinamarca	Cáqueza	N.A.	N.A.	Libertador ^a^	R1, R2	3_Cundinamarca	40_Cundinamarca
Cundinamarca	Quetame	N.A.	N.A.	Nicolas ^b^	R1, R2	4_Cundinamarca	41_Cundinamarca
Cundinamarca	Quetame	N.A.	N.A.	Nicolas ^b^	R1, R2	5_Cundinamarca	42_Cundinamarca
Cundinamarca	Quetame	N.A.	N.A.	Nicolas ^b^	R1, R2	6_Cundinamarca	43_Cundinamarca
Cundinamarca	Quetame	N.A.	N.A.	Nicolas ^b^	R1, R2	7_Cundinamarca	44_Cundinamarca
Cundinamarca	Quetame	N.A.	N.A.	Nicolas ^b^	R1, R2	8_Cundinamarca	45_Cundinamarca
Boyacá	Villa de Leyva	N.A.	N.A.	Aslam ^a^	R1, R2, R3		46_Boyacá
Boyacá	Villa de Leyva	N.A.	N.A.	Aslam ^a^	R1, R2, R4	9_Boyacá	58_Boyacá
Boyacá	Villa de Leyva	N.A.	N.A.	Aslam ^a^	R1, R2, R3	10_Boyacá	47_Boyacá
Boyacá	Villa de Leyva	N.A.	N.A.	Libertador ^a^	R1, R2	11_Boyacá	48_Boyacá
Boyacá	Villa de Leyva	N.A.	N.A.	Roble ^a^	R1, R2, R3	12_Boyacá	49_Boyacá
Boyacá	Villa de Leyva	N.A.	N.A.	Roble ^a^	R1, R2, R3	13_Boyacá	50_Boyacá
Boyacá	Villa de Leyva	N.A.	N.A.	Roblea ^a^	R1, R2, R3	14_Boyacá	71_Boyacá
Boyacá	Villa de Leyva	N.A.	N.A.	Libertador ^a^	R1, R2		117_Boyacá
Boyacá	Sutamarchán	N.A.	N.A.	Libertador ^a^	R1, R2	15_Boyacá	51_Boyacá
Cundinamarca	Mosquera	4°41′44″	74°12′12″	Santa Clara ^a^	N.D.	16_Cundinamarca (Fu40) ^e^	
Boyacá	Santa Sofía	5°43′2.36″	73°36′7.38″	Conquistador ^a^	R1, R2	17_Boyacá	52_Boyacá
Boyacá	Santa Sofía	5°43′15.36″	73°35′28.9″	Monterone ^a^	R1, R2	18_Boyacá	53_Boyacá
Boyacá	Santa Sofía	5°43′10.57″	73°35′41.05″	Nicolás ^b^	R2	19_Boyacá	54_Boyacá
Boyacá	Santa Sofía	5°41′42.72″	73°35′19.73″	Libertador ^a^	R1, R2	20_Boyacá	55_Boyacá
Boyacá	Santa Sofía	5°41′42.72″	73°35′19.73″	Aslam ^a^	R1, R2	21_Boyacá	56_Boyacá
Antioquia	El Peñol	6°15′25.39″	75°16′28.18″	Aslam ^a^	R1, R2, R3	22_Antioquia	57_Antioquia
Antioquia	El Peñol	6°15′25.39″	75°16′28.18″	Aslam ^a^	R1, R2, R3	23_Antioquia	59_Antioquia
Antioquia	El Peñol	N.A.	N.A.	Aslam ^a^	R1, R2, R3	24_Antioquia	72_Antioquia
Antioquia	El Peñol	6°15′25.39″	75°16′28.18″	Gem 604 ^a^	R1, R2, R3	25_Antioquia (Fut31)	60_Antioquia
Antioquia	El Peñol	6°16′04.98″	75°14′54.27″	Aslam ^a^	R1, R2	26_Antioquia	61_Antioquia
Antioquia	El Peñol	6°16′04.98″	75°14′54.27″	Aslam ^a^	R1, R2, R3	27_Antioquia	62_Antioquia
Antioquia	Urrao	6°15′8.41″	75°14′48″	Venanzio ^a^	R1, R2, R3	28_Antioquia	63_Antioquia
Antioquia	Urrao	6°15′48.78″	76°07′39.20″	Venanzio ^a^	N.D.	29_Antioquia	64_Antioquia
Antioquia	Urrao	6°15′48.78″	76°07′39.20″	Torrano ^a^	N.D.	30_Antioquia	65_Antioquia
Antioquia	Urrao	6°16′41.74″	76°05′46.18″	Torrano ^a^	R1, R2	31_Antioquia	66_Antioquia
Antioquia	Urrao	6°16′41.74″	76°05′46.18″	Torrano ^a^	R1, R2	32_Antioquia	67_Antioquia (Fur38) ^g^
Antioquia	Urrao	6°20′38.56″	76°05′30.03″	Torrano ^a^	R1, R2	33_Antioquia	68_Antioquia
Antioquia	Urrao	6°20′38.56″	76°05′30.03″	Torrano ^a^	R1, R2	34_Antioquia	69_Antioquia
Antioquia	Urrao	6°20′38.56″	76°05′30.03″	Torrano ^a^	R1, R2	35_Antioquia	70_Antioquia
Antioquia	Urrao	6°20′29.3″	76°07′62.1″	Torrano ^a^	R1, R2		118_Antioquia
Antioquia	Urrao	6°20′11.5″	76°08′30.50″	Roble ^a^	R1, R2, R3		119_Antioquia
Antioquia	Medellín	6°18′33.3″	75°67′54.54″	Roble ^a^	R1, R2, R3	120_Antioquia (Fut64) ^g^	
Cundinamarca	Fómeque	4°28.094′	73°31.868′	Roble ^a^	R1, R2, R3	73_Cundinamarca	88_Cundinamarca
Cundinamarca	Fómeque	4°28.094′	73°31.868′	Roble ^a^	R1, R2, R3	74_Cundinamarca	89_Cundinamarca
Cundinamarca	Fómeque	4°28.053′	33°51.980′	Libertador ^a^	R1, R2		90_Cundinamarca
Cundinamarca	Fómeque	N.A.	N.A.	Nicolás ^b^	R1, R2	75_Cundinamarca	91_Cundinamarca
Cundinamarca	Fómeque	N.A.	N.A.	Nicolás ^b^	R1, R2	76_Cundinamarca	92_Cundinamarca
Antioquia	Rionegro	6°9.735′	75°24.307′	Tropical ^c^	R1, R2	77_Antioquia	93_Antioquia
Antioquia	Rionegro	6°9.735′	75°24.307′	Tropical ^c^	R1, R2	78_Antioquia	95_Antioquia
Antioquia	Rionegro	6°9.735′	75°24.307′	Tropical ^c^	R1, R2	79_Antioquia	96_Antioquia
Antioquia	Rionegro	6°9.735′	75°24.307′	Tropical ^c^	R1, R2	80_Antioquia	97_Antioquia
Antioquia	Rionegro	6°9.735′	75°24.307′	Tropical ^c^	R1, R2	81_Antioquia (Fut52) ^g^	98_Antioquia
Antioquia	San Vicente	6°16.222′	75°22.361′	N.A.	N.D.	82_Antioquia	99_Antioquia
Antioquia	San Vicente	6°16.222′	75°22.361′	N.A.	N.D.	83_Antioquia	100_Antioquia
Antioquia	San Vicente	6°16.222′	75°22.361′	N.A.	N.D.	84_Antioquia	101_Antioquia (Fur55) ^g^
Antioquia	San Vicente	6° 16.222′	75°22.361′	N.A.	N.D.	85_Antioquia	102_Antioquia
Antioquia	Guarne	N.A.	N.A.	N.A.	N.D.	86_Antioquia	103_Antioquia
Antioquia	Guarne	N.A.	N.A.	N.A.	N.D.	87_Antioquia	104_Antioquia
Caldas	Chinchiná	4° 56′33″	75°37′10″	Calima ^a^	R1, R2	116_Antioquia	94_Caldas (*Fol*59) ^f^
Caldas	Manizales	5°3′14″	75°29′30″	N.A.	N.D.		105_Caldas (*Fol-*UDC10) ^f^
Caldas	Manizales	N.A.	N.A.	N.A.	N.D.		106_Caldas
Caldas	Chinchiná	4°59′7″	75°39′58″	Armada Carguero ^d^	R1, R2		107_Caldas
Caldas	Chinchiná	4°59′7″	75°39′58″	Armada Carguero ^d^	R1, R2		108_Caldas
Caldas	Palestina	4°56′29″	75°39′25″	Roble ^a^	R1, R2, R3		109_Caldas
Caldas	Manizales	5°5′55″	75°37′13″	Venanzio ^a^	N.D.		110_Caldas
Caldas	Villamaría	4°57′297″	75°31.371′	Roble ^a^	R1, R2, R3		111_Caldas
Caldas	Chinchiná	05°00.447′	75°35.831′	Calima ^a^	R1, R2		112_Caldas
Caldas	Villamaría	4° 57′397′	75° 31.377′	Roble ^a^	R1, R2, R3		113_Caldas
Caldas	Chinchiná	04°56′ 34″	75°37′7″	Calima ^a^	R1, R2		114_Caldas
Caldas	Palestina	05°1′ 6″	75°36′40″	Ciénaga ^a^	R1, R2		115_Caldas

^a^ Hybrids derived from the cultivar Chonto. ^b^ Hybrids derived from the variety Milano. ^c^ Hybrids derived from the cultivar Cherry (*Solanum lycopersicum* var. *cerasiforme*). ^d^ Hybrids obtained by grafting. ^e^ *Fusarium oxysporum* reference strain (Fu40) obtained from the microorganism germplasm bank of AGROSAVIA. ^f^ 94_Caldas = *Fol*59 and 105_Caldas = *Fol-*UDC10 (*F. oxysporum* f. sp. *lycopersici*). ^g^ Fungal isolates identified as *Forl* using the new marker (*Forl*_155.3). N.A.: not available. N.D.: not determined. R1 = *Fol* race 1; R2 = *Fol* race 2; R3 = *Fol* race 3.

**Table 2:** Primers used to identify *Fusarium oxysporum* isolates.

Primer	Sequence	Region Amplified	Predicted Size
Uni F	ATCATCTTGTGCCAACTTCAG	*Pg1* ^a^	670 bp ^a^
Uni R	GTTTGTGATCTTTGAGTTGCCA		
EF1-F	ATGGGTAAGGAGGACAAGACTCA	*EF1A* ^b^	776 bp ^b^
EF1-R	TGGAGATACCAGCCTCGAAC		
P12-F2B	GTATCCCTCCGGATTTTGAGC	*SIX1* ^c^	992 bp
P12-R1	AATAGAGCCTGCAAAGCATG		
SIX3-F1	CCAGCCAGAAGGCCAGTTT	*SIX3* ^c^	608 bp ^c^
SIX3-R2	GGCAATTAACCACTCTGCC		
G121A-F2 ^e^	ACGGGGTAACCCATATTGCA		429 bp ^d^
G134A-F2 ^e^	TTGCGTGTTTCCCGGCCA		414 bp ^d^
G137C-F1 ^e^	GCGTGTTTCCCGGCCGCCC		412 bp ^d^
SIX4-F1	TCAGGCTTCACTTAGCATAC	*SIX4* ^c^	967 bp
SIX4-R1	GCCGACCGAAAAACCCTAA		
Forl_155.3F	GGTGAGGTTGCCACATTTCT	GCA_000260155.3 ^f^	337 bp
Forl_155.3R	TCTTTGTTCATTCCCCAAGC		

^a^*Endo-polyglacturonase* gene (*Pg1*) (Ensembl fungi ID: FOXG_14695), reported by Hirano and Arie [32]. ^b^ *Translation elongation factor 1 alpha* gene (*EF1a*) (GenBank: MK172058.1), reported by Cobo-Diaz et al. [30]. ^c^ Reported by van der Does et al. [7]. ^d^ Reported by Lievens et al. [21]. ^e^ Amplicon generated through combination with the SIX3-R2 primer, according to Lievens et al. [21]. ^f^ National Center for Biotechnology Information (NCBI) genome accession number of *Fusarium oxysporum* f. sp. *radicis-lycopersici*.

**Table 3:** Homologous *EF1a* genes in the *Fusarium* species used in the phylogenetic analysis.

GenBank Accession	*Fusarium* Species	Isolate/Code	Reference
KY365602.1	*avenaceum*	UBOCC-A-109 096	[30]
MG550947.1	*verticillioides*	UBOCC-A-109 122	
MH496628.1	*proliferatum*	SL0018	[31]
MK604519.1	*fujikuroi*	SL0089	
KY283870.1	*graminearum*	17a	GenBank
KX034250.1	*falciforme*	RRK20	[33]
MG857253.1	*solani*	SL0002	[31]
MG707130.1	*solani*	SL0016	
MH716809.1	*commune*	SL0021	
JF740825.1	*foetens*	FOSC-21	
AB917000.1	*Fusarium* sp.	SL0584	GenBank
AB817202.1	*Fusarium* sp.	FOSC-1065	
***Fusarium oxysporum* Species Complex**			
MALH01000103.1	f. sp. *lycopersici* race 1	Fol004	[34]
MALV01000081.1	f. sp. *lycopersici* race 2	Fol4287	
RBXW01000019.1	f. sp. *lycopersici* race 3	D11	GenBank
FOCG_09093	f. sp. *radicis-lycopersici*	26381	Ensembl fungi
FOZG_08502	*oxysporum*	Fo047	
JX258849.1	*oxysporum*	Bra2	GenBank
MK172058.1	*oxysporum*	FR3	
MK226334.1	*oxysporum*	SL0001	[31]
MK414772.1	*oxysporum*	SL0035	
MK799835.1	*oxysporum*	SL0019	

## Supplementary Materials

The following are available online at https://www.mdpi.com/2076-0817/9/1/70/s1, Figure S1: Morphological propagative structures of the isolate *Fol*-UDC10, observed on CDA plates; Figure S2: Polymerase chain reaction (PCR) analysis showing the presence of the fragment of the *pg1* gene specific to *Fusarium oxysporum*; Figure S3: PCR analysis showing the presence of the *EF1a* gene on 113 from the 120 isolates obtained; Figure S4: PCR results for *SIX1*, *SIX4*, and *SIX3* for the 120 fungal isolates; Figure S5: Disease scale implemented in this study; Figure S6: Symptom development of *Fusarium oxysporum* f. sp. *radicis-lycopersici* (*Forl*)-infected tomato cultivar (Santa Cruz Kada); Table S1: GenBank sequence unique identifiers and annotation information for the *EF1a* gene; Table S2: Disease scale implemented in this study.
