# Supplementary material for: Characterization of Pathogenic and Nonpathogenic Fusarium oxysporum Isolates Associated with Commercial Tomato Crops in the Andean Region of Colombia
Source: Pathogens. 2020 Jan 20;9(1):70. doi: 10.3390/pathogens9010070 (PMC7168637; doi:10.3390/pathogens9010070)
Supplement: Supplementary file 1 [file pathogens-09-00070-s001.zip › Supplementary Figure 1.pptx]

## Slide 1
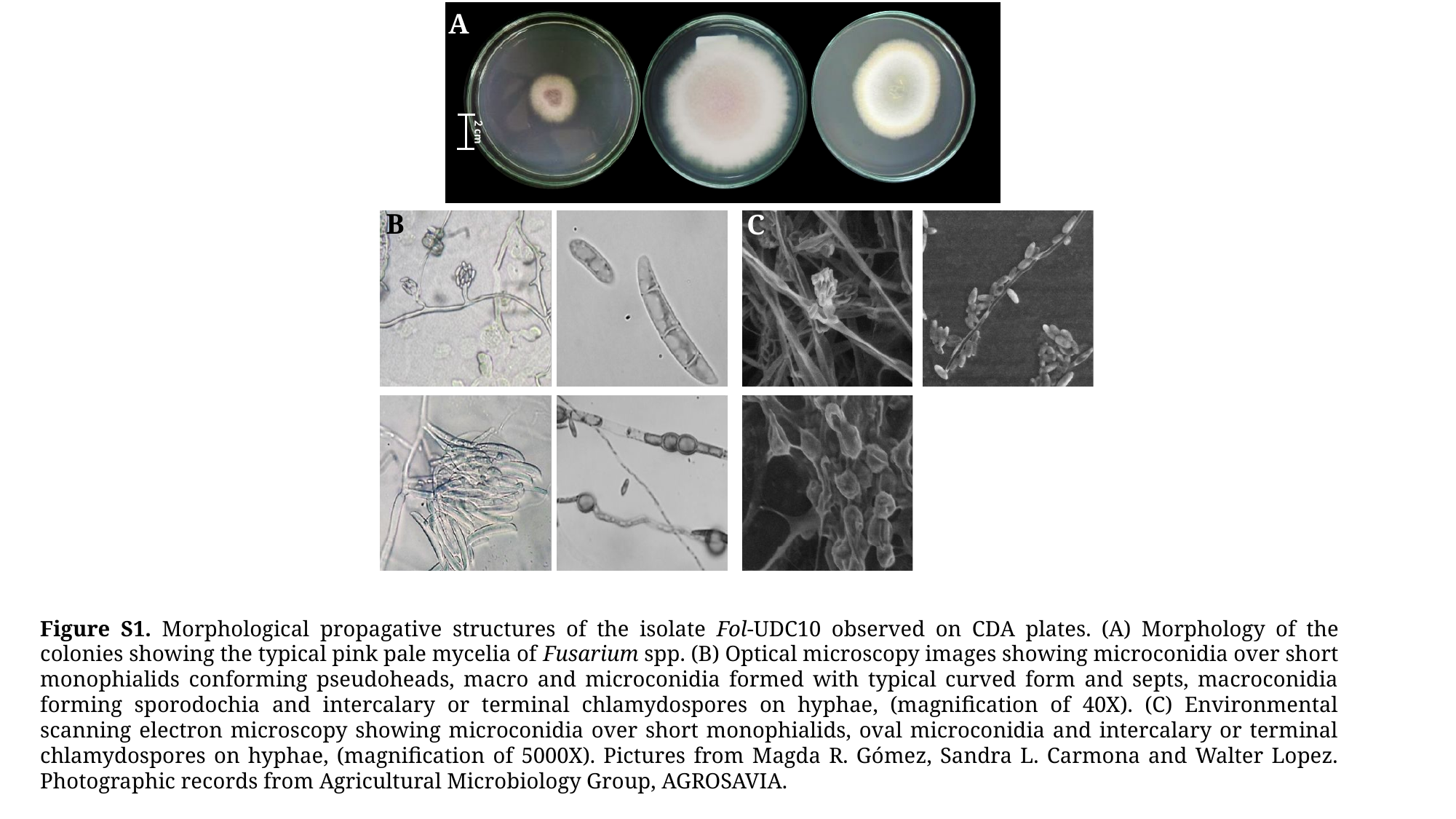

2 cm
A
B
C
Figure S1. Morphological propagative structures of the isolate Fol-UDC10 observed on CDA plates. (A) Morphology of the colonies showing the typical pink pale mycelia of Fusarium spp. (B) Optical microscopy images showing microconidia over short monophialids conforming pseudoheads, macro and microconidia formed with typical curved form and septs, macroconidia forming sporodochia and intercalary or terminal chlamydospores on hyphae, (magnification of 40X). (C) Environmental scanning electron microscopy showing microconidia over short monophialids, oval microconidia and intercalary or terminal chlamydospores on hyphae, (magnification of 5000X). Pictures from Magda R. Gómez, Sandra L. Carmona and Walter Lopez. Photographic records from Agricultural Microbiology Group, AGROSAVIA.
