# Supplementary material for: Characterization of Pathogenic and Nonpathogenic Fusarium oxysporum Isolates Associated with Commercial Tomato Crops in the Andean Region of Colombia
Source: Pathogens. 2020 Jan 20;9(1):70. doi: 10.3390/pathogens9010070 (PMC7168637; doi:10.3390/pathogens9010070)
Supplement: Supplementary file 1 [file pathogens-09-00070-s001.zip › Supplementary Figure 2.pptx]

## Slide 1
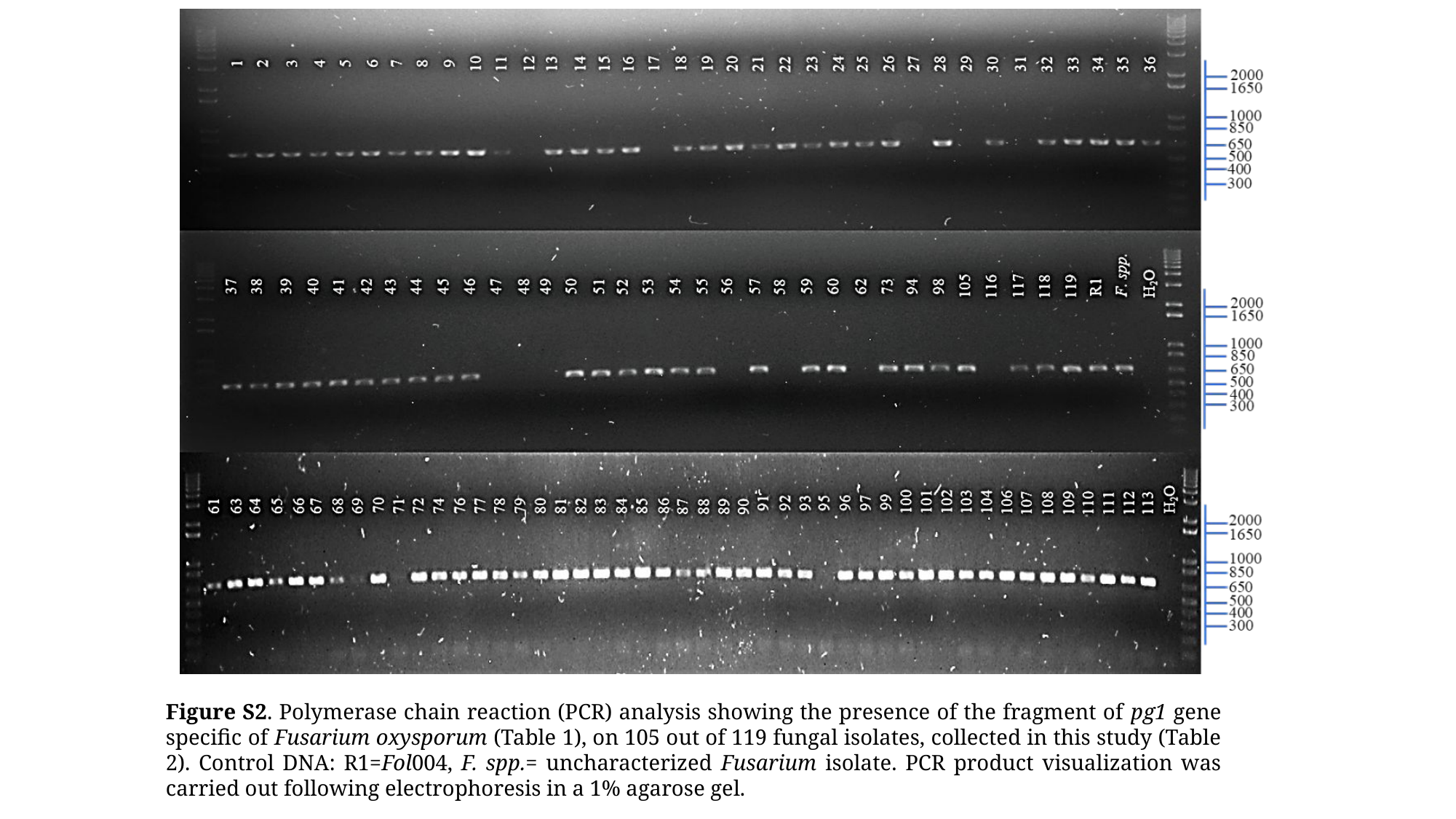

Figure S2. Polymerase chain reaction (PCR) analysis showing the presence of the fragment of pg1 gene specific of Fusarium oxysporum (Table 1), on 105 out of 119 fungal isolates, collected in this study (Table 2). Control DNA: R1=Fol004, F. spp.= uncharacterized Fusarium isolate. PCR product visualization was carried out following electrophoresis in a 1% agarose gel.
