# Supplementary material for: Characterization of Pathogenic and Nonpathogenic Fusarium oxysporum Isolates Associated with Commercial Tomato Crops in the Andean Region of Colombia
Source: Pathogens. 2020 Jan 20;9(1):70. doi: 10.3390/pathogens9010070 (PMC7168637; doi:10.3390/pathogens9010070)
Supplement: Supplementary file 1 [file pathogens-09-00070-s001.zip › Supplementary Figure 3.pptx]

## Slide 1
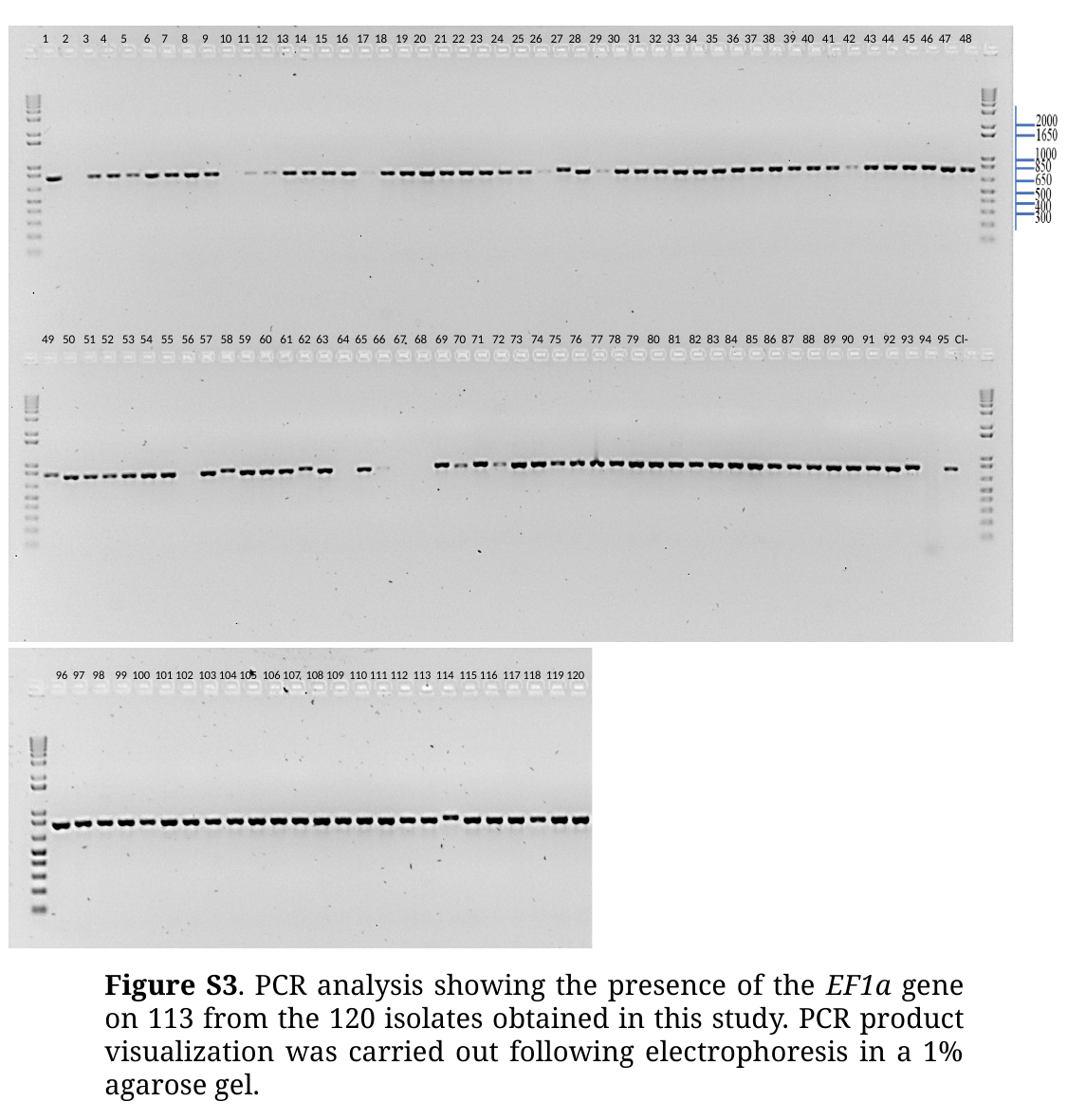

1 2 3 4 5 6 7 8 9 10 11 12 13 14 15 16 17 18 19 20 21 22 23 24 25 26 27 28 29 30 31 32 33 34 35 36 37 38 39 40 41 42 43 44 45 46 47 48
49 50 51 52 53 54 55 56 57 58 59 60 61 62 63 64 65 66 67 68 69 70 71 72 73 74 75 76 77 78 79 80 81 82 83 84 85 86 87 88 89 90 91 92 93 94 95 Cl-
96 97 98 99 100 101 102 103 104 105 106 107 108 109 110 111 112 113 114 115 116 117 118 119 120
Figure S3. PCR analysis showing the presence of the EF1a gene on 113 from the 120 isolates obtained in this study. PCR product visualization was carried out following electrophoresis in a 1% agarose gel.
