# Supplementary material for: Characterization of Pathogenic and Nonpathogenic Fusarium oxysporum Isolates Associated with Commercial Tomato Crops in the Andean Region of Colombia
Source: Pathogens. 2020 Jan 20;9(1):70. doi: 10.3390/pathogens9010070 (PMC7168637; doi:10.3390/pathogens9010070)
Supplement: Supplementary file 1 [file pathogens-09-00070-s001.zip › Supplementary Figure 4.pptx]

## Slide 1
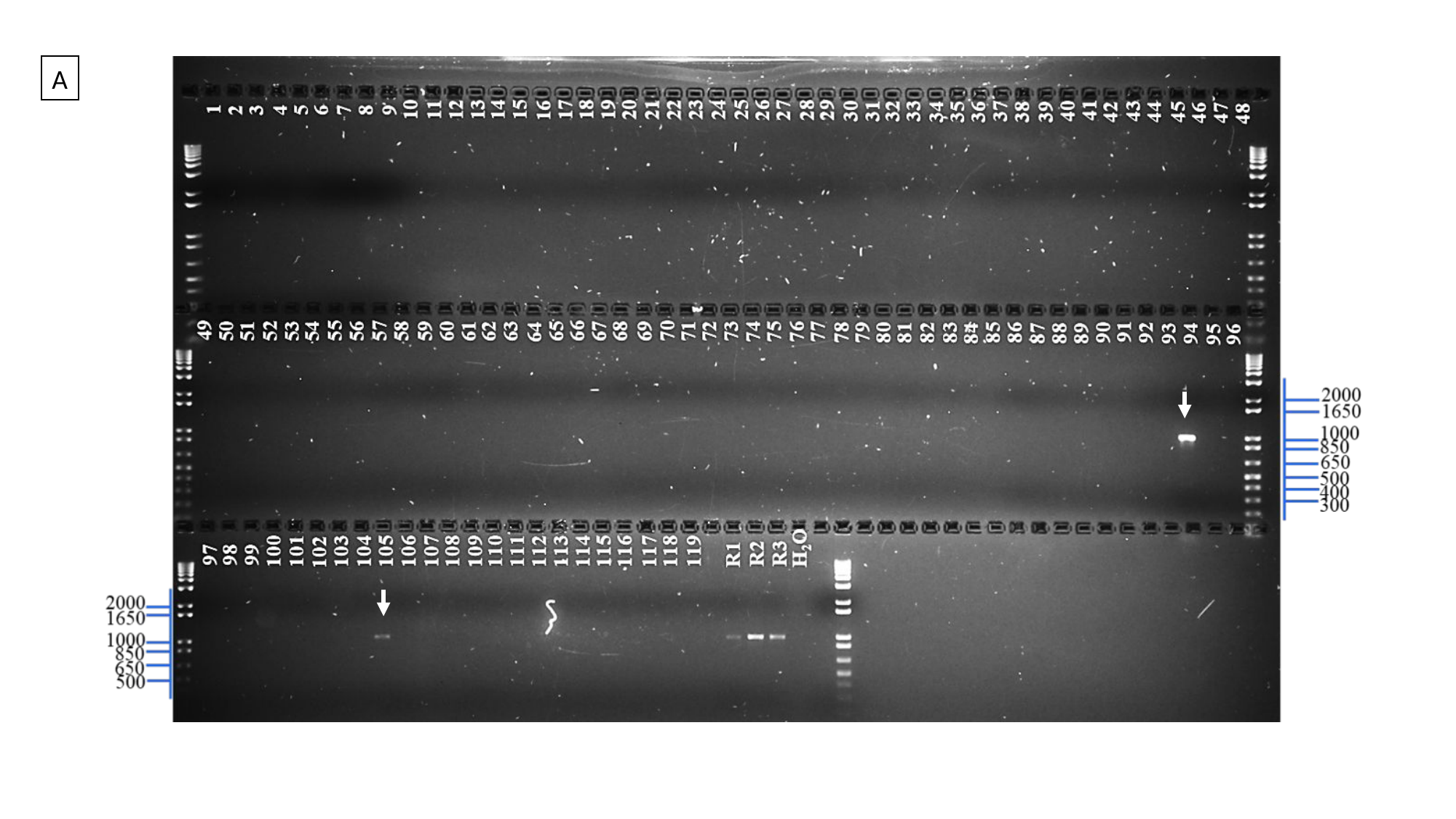

A

## Slide 2
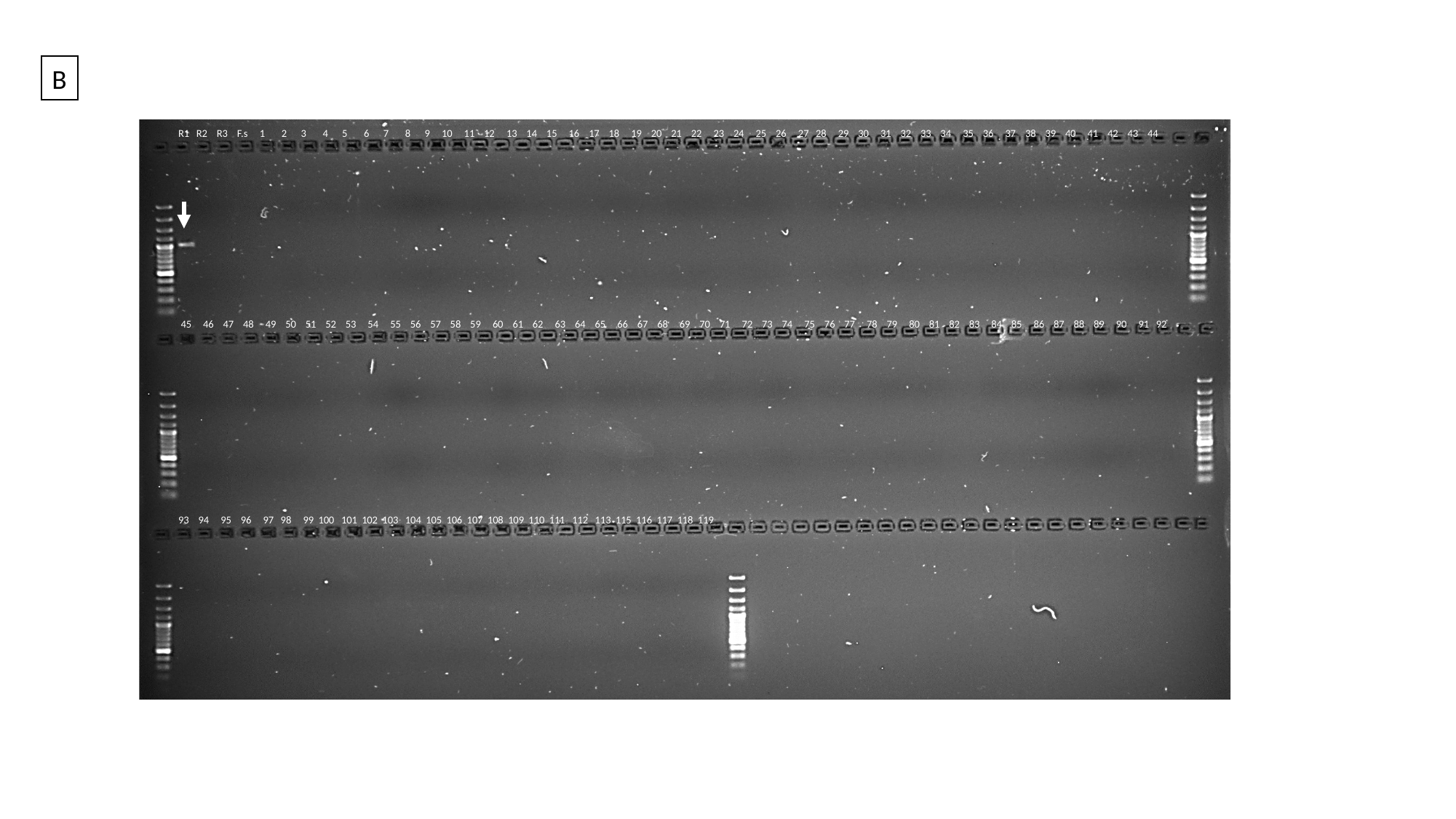

B
R1 R2 R3 F.s 1 2 3 4 5 6 7 8 9 10 11 12 13 14 15 16 17 18 19 20 21 22 23 24 25 26 27 28 29 30 31 32 33 34 35 36 37 38 39 40 41 42 43 44
45 46 47 48 49 50 51 52 53 54 55 56 57 58 59 60 61 62 63 64 65 66 67 68 69 70 71 72 73 74 75 76 77 78 79 80 81 82 83 84 85 86 87 88 89 90 91 92
93 94 95 96 97 98 99 100 101 102 103 104 105 106 107 108 109 110 111 112 113 115 116 117 118 119

## Slide 3
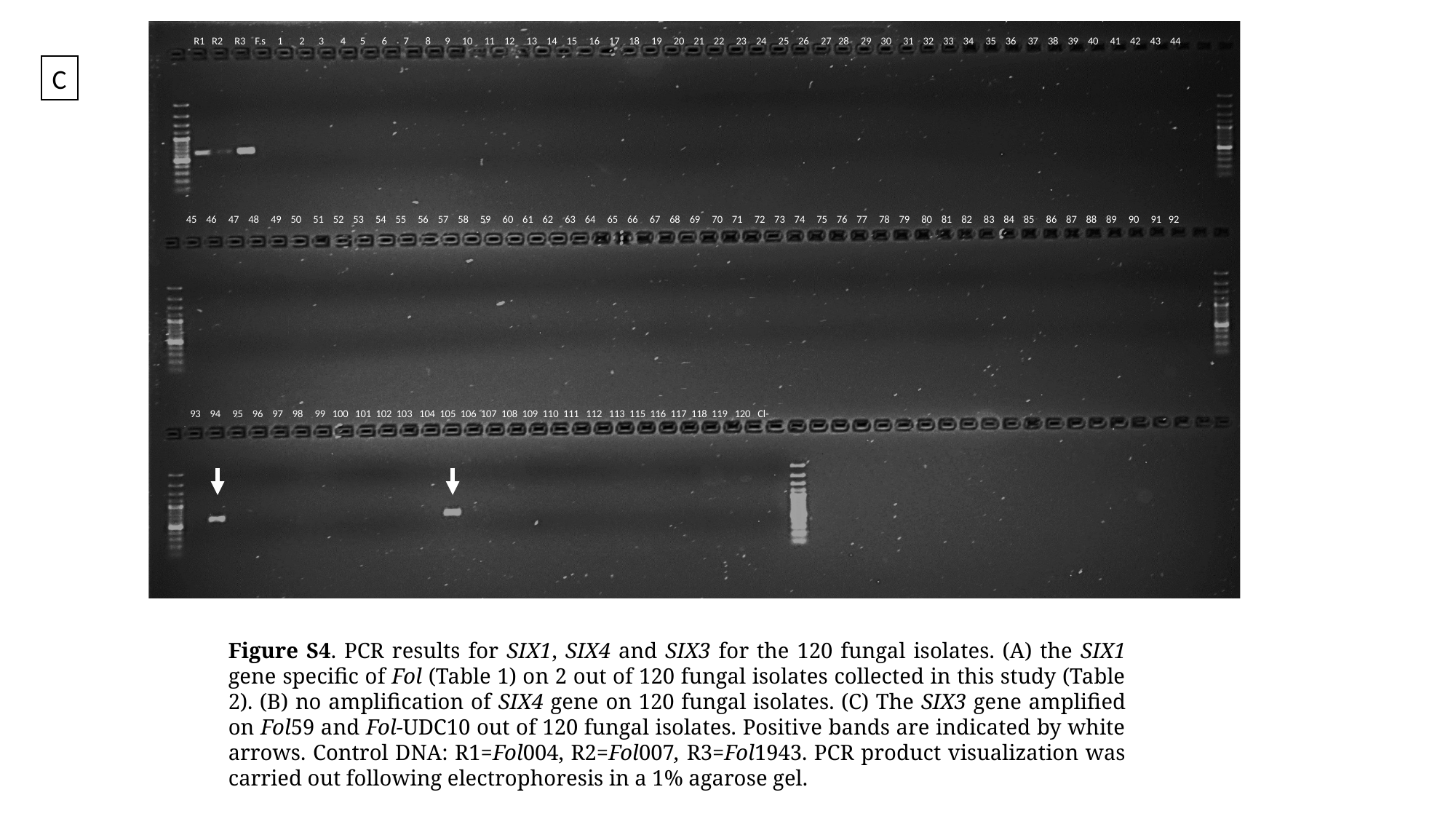

R1 R2 R3 F.s 1 2 3 4 5 6 7 8 9 10 11 12 13 14 15 16 17 18 19 20 21 22 23 24 25 26 27 28 29 30 31 32 33 34 35 36 37 38 39 40 41 42 43 44
C
45 46 47 48 49 50 51 52 53 54 55 56 57 58 59 60 61 62 63 64 65 66 67 68 69 70 71 72 73 74 75 76 77 78 79 80 81 82 83 84 85 86 87 88 89 90 91 92
93 94 95 96 97 98 99 100 101 102 103 104 105 106 107 108 109 110 111 112 113 115 116 117 118 119 120 Cl-
Figure S4. PCR results for SIX1, SIX4 and SIX3 for the 120 fungal isolates. (A) the SIX1 gene specific of Fol (Table 1) on 2 out of 120 fungal isolates collected in this study (Table 2). (B) no amplification of SIX4 gene on 120 fungal isolates. (C) The SIX3 gene amplified on Fol59 and Fol-UDC10 out of 120 fungal isolates. Positive bands are indicated by white arrows. Control DNA: R1=Fol004, R2=Fol007, R3=Fol1943. PCR product visualization was carried out following electrophoresis in a 1% agarose gel.
