# Supplementary material for: Characterization of Pathogenic and Nonpathogenic Fusarium oxysporum Isolates Associated with Commercial Tomato Crops in the Andean Region of Colombia
Source: Pathogens. 2020 Jan 20;9(1):70. doi: 10.3390/pathogens9010070 (PMC7168637; doi:10.3390/pathogens9010070)
Supplement: Supplementary file 1 [file pathogens-09-00070-s001.zip › Supplementary Figure 5.pptx]

## Slide 1
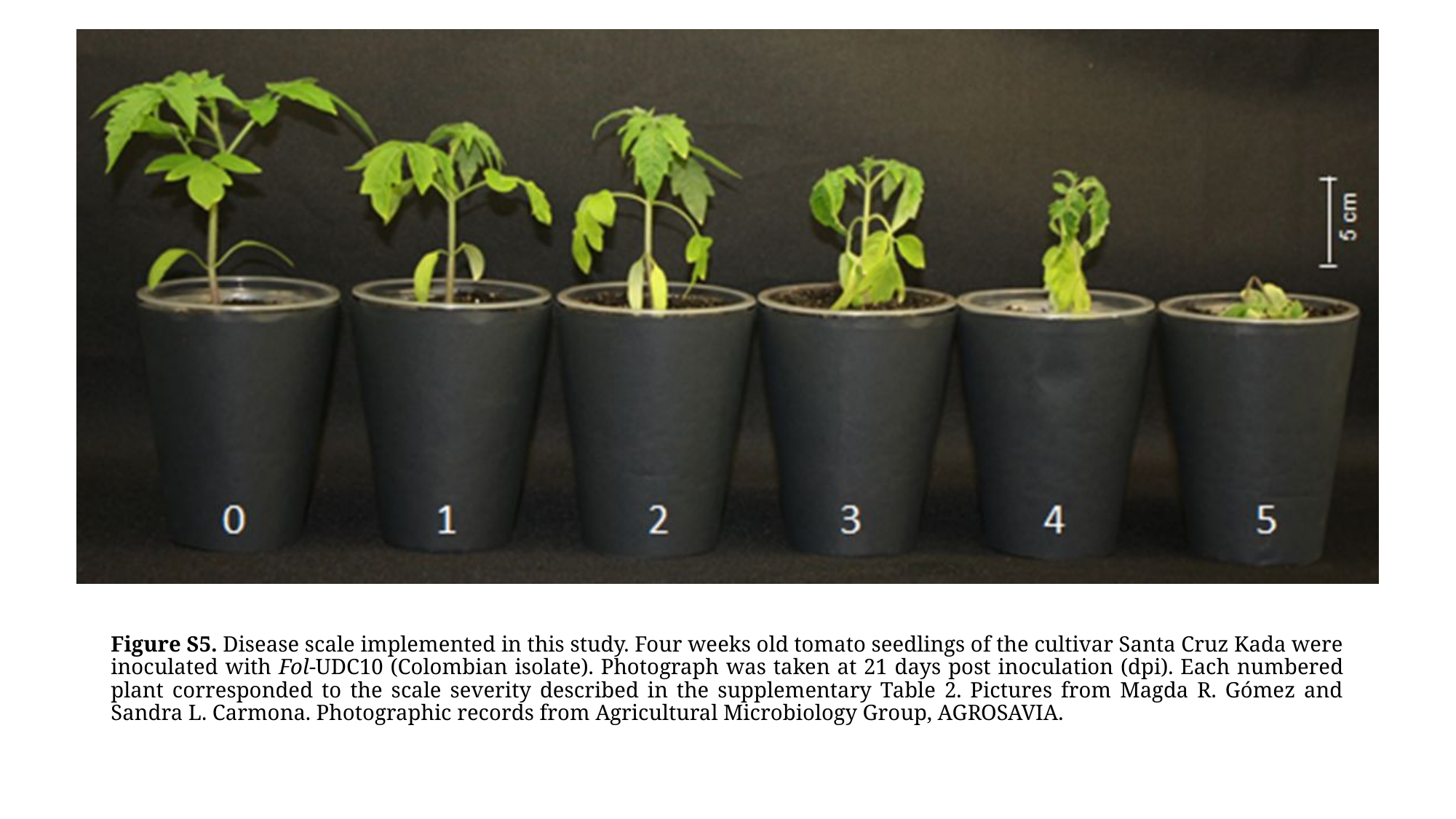

Figure S5. Disease scale implemented in this study. Four weeks old tomato seedlings of the cultivar Santa Cruz Kada were inoculated with Fol-UDC10 (Colombian isolate). Photograph was taken at 21 days post inoculation (dpi). Each numbered plant corresponded to the scale severity described in the supplementary Table 2. Pictures from Magda R. Gómez and Sandra L. Carmona. Photographic records from Agricultural Microbiology Group, AGROSAVIA.
