# Supplementary material for: Characterization of Pathogenic and Nonpathogenic Fusarium oxysporum Isolates Associated with Commercial Tomato Crops in the Andean Region of Colombia
Source: Pathogens. 2020 Jan 20;9(1):70. doi: 10.3390/pathogens9010070 (PMC7168637; doi:10.3390/pathogens9010070)
Supplement: Supplementary file 1 [file pathogens-09-00070-s001.zip › Supplementary Figure 6.pptx]

## Slide 1
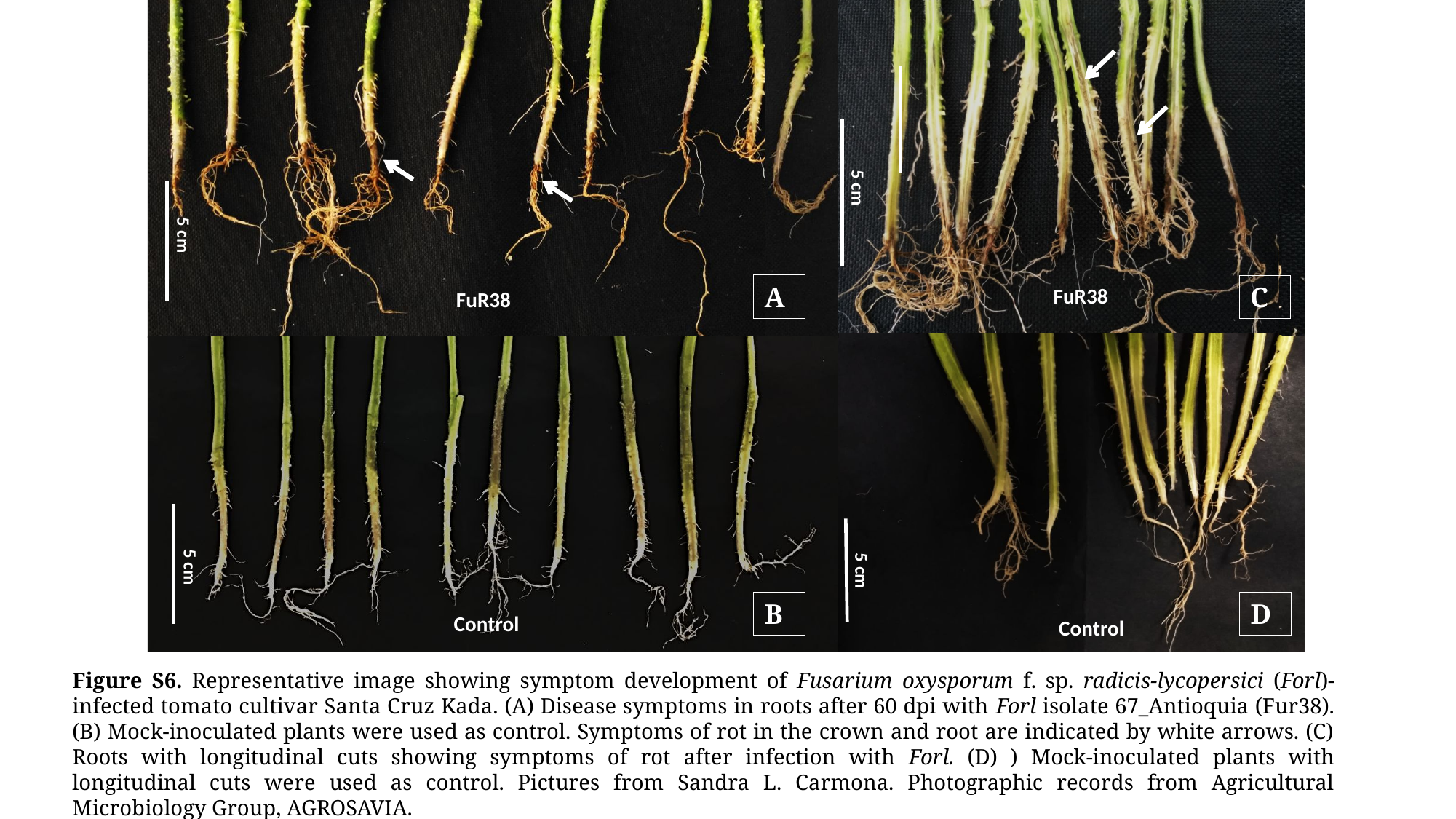

5 cm
5 cm
FuR38
FuR38
5 cm
5 cm
Control
Control
A
C
B
D
Figure S6. Representative image showing symptom development of Fusarium oxysporum f. sp. radicis-lycopersici (Forl)-infected tomato cultivar Santa Cruz Kada. (A) Disease symptoms in roots after 60 dpi with Forl isolate 67_Antioquia (Fur38). (B) Mock-inoculated plants were used as control. Symptoms of rot in the crown and root are indicated by white arrows. (C) Roots with longitudinal cuts showing symptoms of rot after infection with Forl. (D) ) Mock-inoculated plants with longitudinal cuts were used as control. Pictures from Sandra L. Carmona. Photographic records from Agricultural Microbiology Group, AGROSAVIA.
