# Supplementary material for: Characterization of Pathogenic and Nonpathogenic Fusarium oxysporum Isolates Associated with Commercial Tomato Crops in the Andean Region of Colombia
Source: Pathogens. 2020 Jan 20;9(1):70. doi: 10.3390/pathogens9010070 (PMC7168637; doi:10.3390/pathogens9010070)
Supplement: Supplementary file 1 [file pathogens-09-00070-s001.zip › Supplementary Table 2_disease scale.docx]

**Supplementary Table 1**. Disease scale implemented in this study, adapted from (Çakır *et al*., 2014; Rongai *et al*., 2016 and Akhter *et al*., 2015)

| **Symptom** | **Scale** | **Phenotypes** | |
| --- | --- | --- | --- |
|  |  | **Tomato** | ***Fol*** |
| Symptomelss/healthy plant | 0 | Resistant | Non-pathogenic |
| Slight chlorosis in one or two leaves | 1 |  |  |
| Chlorotic cotyledons and few wilted leaves | 2 |  | Weakly virulent |
| Severe chlorosis on leaves, half of the leaves are wilted | 3 | Susceptible | Virulent |
| All the leaves are chlorotic, severe growth distortion | 4 |  | Highly virulent |
| Dead plant​ | 5 |  |  |
